# Inter-Eye Molecular Discrepancies in the Corneal Epithelium Point to *TFRC* in the Keratoconus Severity Signature and Mechanism of Cone Formation

**DOI:** 10.1167/iovs.66.13.52

**Published:** 2025-10-29

**Authors:** Katarzyna Jaskiewicz-Rajewicz, Alicja Wysocka, Magdalena Maleszka-Kurpiel, Eliza Matuszewska-Mach, Jakub Wozniak, Rafal Ploski, Jan Matysiak, Malgorzata Rydzanicz, Marzena Gajecka

**Affiliations:** 1 Institute of Human Genetics, Polish Academy of Sciences, Poznan, Poland; 2 Optegra Eye Health Care Clinic in Poznan, Poland; 3 Poznan University of Medical Sciences, Chair of Ophthalmology and Optometry, Poznan, Poland; 4 Poznan University of Medical Sciences, Chair and Department of Inorganic and Analytical Chemistry, Poznan, Poland; 5 Poznan University of Medical Sciences, Chair and Department of Genetics and Pharmaceutical Microbiology, Poznan, Poland; 6 Department of Genetics and Animal Breeding, Poznan University of Life Sciences, Poznan, Poland; 7 Department of Medical Genetics, Medical University of Warsaw, Warsaw, Poland

**Keywords:** cornea, corneal epithelium, keratoconus severity, inter-eye asymmetry, transcriptomics, proteomics, iron, oxidative stress

## Abstract

**Purpose:**

There is no molecular evidence available confirming or contradicting inter-eye molecular variability in keratoconus (KTCN). The key question is whether the use of a model of more and less affected eyes of the same pair facilitates the identification of specific molecular features of KTCN severity.

**Methods:**

This retrospective case–control study involved 21 KTCN patients (*n* = 132 experimental samples derived from 42 corneal epithelium samples separated into *central*, *middle*, and *peripheral topographic regions*) analyzed in the paired model. Transcriptomic (RNA-sequencing) and proteomic (MALDI-TOF/TOF MS/MS) profiling was performed. An additional non-paired model, including KTCN patients (*n* = 42) and controls (*n* = 14), strengthened the assessment. Then, key findings were validated in a rediscovery study with 20 patients using reverse-transcription quantitative polymerase chain reaction, immunofluorescence staining, and confocal microscopy.

**Results:**

In the paired model, which included patients with ≥1 grade difference in topographic keratoconus classification, 48 differentially expressed genes were identified. Over-representation analysis highlighted the GO term “cell–cell adhesion” (adjusted *P* = 0.028), with key contributors including *ACTN1*, *EPCAM*, *PCDH19*, *PVR*, and *TFRC*. *TFRC* showed significantly higher expression in the *middle topographic region* of more severely affected eyes (*P* = 0.008, paired *t*-test), correlating with the average *topographic region* thickness (*R* = 0.53, *P* = 0.008) and flat keratometry (K1; *R* = 0.49, *P* = 0.016). Immunofluorescence confirmed intra-individual and regional differences in transferrin receptor (TFRC) protein expression, with increased expression in the *middle topographic region* of more advanced eyes, compared to eyes with forme fruste KTCN.

**Conclusions:**

Inter-eye variability implicates *TFRC* as a component of the KTCN severity signature, thus adding the element to the mechanisms underlying KTCN cone formation.

Keratoconus (KTCN) is a corneal ectatic disease in which the cornea progressively thins and deforms. In the place of the greatest thinning, due to an increasing steepening, a cone is formed, which is usually located outside the axis of vision. The emerging changes in the cornea lead to protrusions and scars and ultimately to the loss of corneal transparency.^1^ The prevalence of KTCN in the worldwide population is greater than 1 per 2000 individuals; however, ethnicity has been found to play a role in disease occurrence, as well as both recently implemented diagnostic criteria and newer diagnostic devices.^2^^,^^3^

The etiology of KTCN is multifaceted and still not fully understood. Alongside genetic factors, such as Down syndrome and Ehlers–Danlos syndrome, environmental risk factors, including frequent eye rubbing, ocular allergies, and asthma, have been reported.^4^ In addition to studies performed to identify hereditary genetic variants in KTCN, molecular research focusing on RNA and protein molecules has provided insight into the pathophysiology of the disease.^5^^–^^18^ These studies have revealed disturbances in collagen synthesis and maturation, as well as decreased expression of TGF-β, Hippo, and Wnt signaling pathways, all of which are presumed to impact the stromal corneal organization.^18^ Moreover, also reported were disturbances in the corneal epithelium (CE), a subject of this study, such as reduced expression of cell adhesion– and cell integrity–related molecules compromising cell–cell interactions essential for maintaining the stability of the corneal structure,^19^^,^^20^ as well as the imbalance of oxidative stress markers.^7^^,^^16^^,^^17^^,^^21^^,^^22^ Some of these reported molecular changes were more specific to the cone region of the CE,^7^^,^^16^ reflecting both the characteristic for KTCN *doughnut* pattern (thin cone center surrounded by the thickened annulus), as well as the regional physiology of CE differentiation of corneal limbal epithelial stem cells into transient amplifying cells and centripetal movement from the limbal basal layer to the central CE.^23^

Although KTCN is classified as a bilateral condition, it is not uncommon for one eye to be more severely affected than the other. This asymmetry can be assessed using clinical parameters such as keratometry, pachymetry, and posterior corneal elevation values,^24^ as well as manifest refraction.^25^ According to the global consensus, true unilateral KTCN does not exist, and unilateral clinical presentation may occur in a predisposed individual due to asymmetric environmental factors, such as habitual eye rubbing.^26^

The unpredictable rate of progression and ultimate severity of KTCN pose clinical and therapeutic challenges. The corneal tomography (or combined tomography and topography) is commonly used in KTCN diagnosis and monitoring its progression, whereas maximal corneal curvature (K_max_) is the most often reported as the best single indicator for detecting KTCN progression.^27^^–^^29^ However, no uniform criteria for defining KTCN progression have yet been developed.^29^ Also, in detecting early-stage KTCN, the epithelium thickness mapping could be useful as the epithelial thickness irregularity and asymmetry are discriminating factors between keratoconic and healthy eyes.^30^ Because of these clinical findings and implications, the phenotypic changes observed in CE and the accompanying molecular alterations have become a subject of scientific interest.

To date, no molecular data or findings have been reported to indicate or refute molecular variability between pairs of eyes in patients with KTCN. Given the heterogeneity of KTCN, the intra-individual comparisons between the more and less affected eyes can provide a valuable approach to uncovering molecular features associated with KTCN severity (possible progression). Here we compared the transcriptomic and proteomic profiles of CE derived from both eyes of the same patients, additionally taking into account the previously defined corneal *topographic regions* that cover the CE *doughnut* pattern,^7^ and we characterized both clinical and molecular features of the inter-eye asymmetry. Identification of specific and sensitive molecular indicators of disease severity could improve the monitoring of KTCN patients and guide the implementation of surgical treatments when necessary.

## Materials and Methods

### Patients, Control Individuals, and Clinical Evaluation

The study protocol was approved by the Institutional Review Board at Poznan University of Medical Sciences, Poznan, Poland (decisions no. 453/14, 755/19, and 180/24). Informed consent was obtained from all participants, according to the tenets of the Declaration of Helsinki. All patients, in both discovery and rediscovery studies, were recruited at the Optegra Eye Health Care Clinic in Poznan, Poland, between October 2019 and December 2024.

Each patient with KTCN and control individual underwent a complete ophthalmological examination, including corneal tomography and epithelial thickness mapping, as previously described.^7^ KTCN was diagnosed based on corneal tomography with a rotating Scheimpflug camera, WaveLight Oculyzer II (Alcon, Fort Worth, TX, USA) or Pentacam (OCULUS, Wetzlar, Germany); epithelial thickness mapping within the 8-mm diameter with spectral-domain optical coherence tomography (SD-OCT) devices, either the CIRRUS 5000 (Carl Zeiss Meditec, Jena, Germany) or MS-39 (Costruzione Strumenti Oftalmici, Florence, Italy); and by slit-lamp and dilated funduscopic examinations. KTCN grade classification was evaluated with respect to the Belin ABCD keratoconus staging system, using topographic keratoconus classification (TKC) derived from the Pentacam (software versions 1.22r09 and 1.26r28).^31^

The inclusion and exclusion criteria for patients with KTCN and control individuals (mild myopia patients) were consistent with the previously established criteria in our KTCN research.^7^^,^^18^^,^^32^ Only patients with KTCN classified for treatment of KTCN with the corneal cross-linking (CXL) procedure were included. For each patient with KTCN, more and less affected eyes were defined, according to TKC, keratometry, posterior elevation, anterior elevation, thinnest corneal thickness (TCT), and manifest refraction. For each pair of control eyes, categorization as more affected and less affected eyes was also performed, according to keratometry, posterior elevation, anterior elevation, TCT, and manifest refraction. JASP software^33^ was used in the statistical evaluation of clinical features and parameters as described in Supplementary Methods S1.1. Here, the discovery and rediscovery studies were designed, and two analysis models, the paired and non-paired, were applied, as explained in the following.

### Material Collection and Sample Preparation

Stamps were made on the CE corresponding to the nose and eyebrow before excision during the CXL and photorefractive keratectomy (PRK) procedures. The obtained tissues were immediately submerged in RNA stabilization solution (RNAlater; Qiagen, Hilden, Germany) after excision and stored at −80°C until the isolation of DNA, RNA, and proteins. The procedure for designating the specific *topographic regions* of the CE was consistent with our previous study.^7^

### RNA and Protein Extraction

Total RNA, DNA, and proteins were extracted from CE samples using the RNA/DNA/Protein Purification Plus Micro Kit (Norgen Biotek, Thorold, ON, Canada), as previously described,^7^ including DNase I RNase-free treatment (EURx, Gdansk, Poland) and RNA quality and quantity assessment using the RNA 6000 Nano Kit (Agilent Technologies, Santa Clara, CA, USA) and NanoDrop Spectrophotometer ND-1000 (NanoDrop Technologies, Wilmington, DE, USA). The quality and quantity of the purified protein samples were evaluated using an Agilent Protein 230 Kit. The RNA and protein samples were stored at −80°C until further analysis.

### Total RNA Library Preparation and Sequencing and RNA-Sequencing Data Analyses

Total RNA libraries were prepared using the TruSeq Stranded Total RNA Library Prep Gold (Illumina, San Diego, CA, USA) according to the manufacturer's protocol. A 100-bp paired-end sequencing run was conducted on the Illumina NovaSeq 6000 platform. The CE samples were sequenced with an average coverage of 100 million read pairs per sample.

Raw reads were trimmed using the BBDuk2 program from the BBTools suite (http://jgi.doe.gov/data-and-tools/bbtools/) to remove Illumina adapters, low-quality regions (mean Phred quality < 5), and reads matching human ribosomal RNA (rRNA) sequences. Kallisto, assisted by GENCODE 34 (Ensembl 100) annotations, was used to estimate transcript expression values. Differential expression analysis was performed in three settings: (1) all patients with KTCN; (2) patients with KTCN whose inter-eye asymmetry expressed in the TKC grades was ≥1 (TKC difference ≥ 1; *n* = 6 pairs); and (3) patients with KTCN whose inter-eye asymmetry expressed in the TKC grades was >1 (TKC difference > 1; *n* = 5 pairs). For each *topographic region* (*central*, *middle*, or *peripheral*) and each setting, analysis was performed separately. *P* values were adjusted for multiple comparisons using the Benjamini–Hochberg correction method in the limma package.^34^^,^^35^ Genes were considered differentially expressed based on the following cutoffs: false discovery rate (FDR) < 0.05 and absolute log_2_-transformed fold change (|log_2_FC|) >0.5.

To create the heatmaps, the data were normalized using library size factors and log transformation with the scuttle package.^36^ Following normalization, we scaled the log-transformed counts to standardize the data, using the scale function in the R environment version 4.4.0.^37^ The heatmaps were then generated using the pheatmap package,^38^ with hierarchical clustering performed using Euclidean distance. Over-representation analysis using ConsensusPathDB^39^ was performed to identify biological pathways/processes or gene sets that were significantly overrepresented in the given gene list. In the analysis, the provided gene set and a collection of biological pathways-based and Gene Ontology (GO)-based sets were compared, followed by statistical significance calculations to determine which pathways were overrepresented. The *P* values were corrected for multiple testing using the FDR method (*q*-values), and *P* < 0.05 was considered statistically significant.

### Tandem Matrix-Assisted Laser Desorption/Ionization Time-of-Flight/Time-of-Flight Mass Spectrometry Protein–Peptide Profiling

Sample preparation, including in-solution tryptic digestion overnight, for tandem matrix-assisted laser desorption/ionization time-of-flight/time-of-flight mass spectrometry (MALDI-TOF/TOF MS) analysis was conducted as previously described.^7^^,^^40^ The analysis was performed using an UltrafleXtreme mass spectrometer (Bruker, Billerica, MA, USA) in reflectron mode, with a mass-to-charge ratio (*m*/*z*) range of 700 to 3500. Proteomic identification was performed based on the SwissProt protein sequence database. Statistical analyses were performed in three settings: (1) all patients with KTCN (*n* = 36 pairs), (2) patients with KTCN whose inter-eye asymmetry expressed in the TKC grades was ≥1 (TKC difference ≥ 1; *n* = 8 pairs), and (3) patients with KTCN whose inter-eye asymmetry expressed in the TKC grades was >1 (TKC difference > 1; *n* = 7 pairs). The analyses were carried out using JASP software,^33^ and further details are provided in Supplementary Methods S1.2. The set of dysregulated proteins was analyzed in overrepresentation analysis and visualized using ShinyGO.^41^

### RNA-Sequencing Data Validation in the Rediscovery Study Using Reverse-Transcription Quantitative Polymerase Chain Reaction

**Figure 1.**
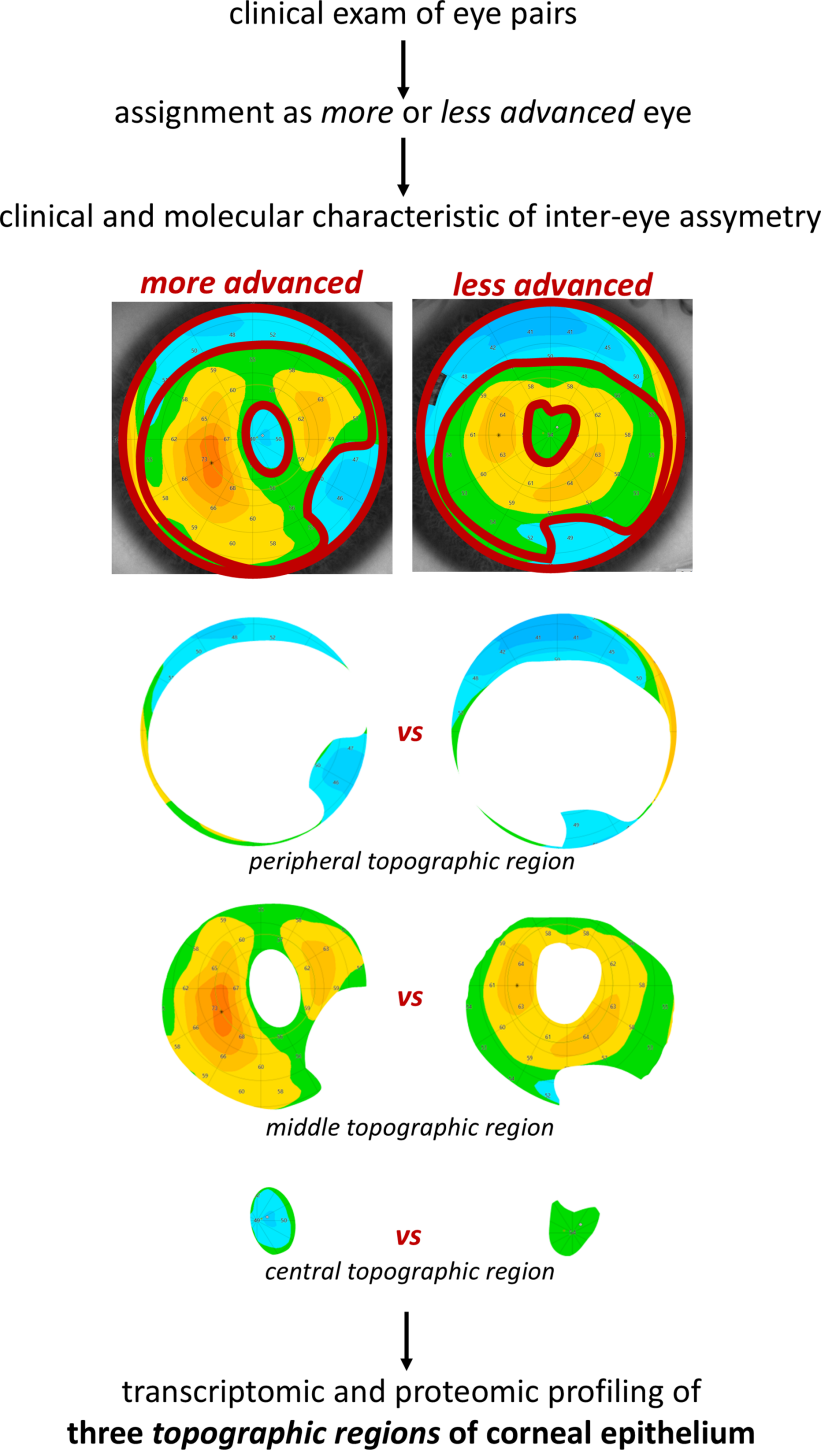
**Study workflow.** First, each patient diagnosed with KTCN underwent a complete ophthalmological examination, including corneal tomography and epithelial thickness mapping of both eyes. Next, more and less advanced eyes were assigned, and simultaneously *central*, *middle*, and *peripheral topographic regions* were designated according to our previously developed experimental model.^7^ CE samples were collected during the CXL procedure, and after the separation of *topographic regions* RNA and protein extraction was performed for high-throughput analyses. The obtained clinical and molecular data were used for paired-samples analysis to check the inter-eye asymmetry in KTCN. Control samples from mild myopia individuals undergoing the PRK procedure were processed in the same manner.

To validate the study results, additional pairs of CE samples were collected and processed according to the study workflow (Fig. 1), followed by reverse-transcription quantitative polymerase chain reaction (RT-qPCR) reactions as described in Supplementary Methods S1.3. Primer sequences and annealing temperatures are listed in Supplementary Table S1.

### Immunofluorescence Staining of CE Samples

The CE samples were fixed with methanol and incubated with primary antibodies, including transferrin receptor (TFRC) antibody (13-6800, Invitrogen), ferritin light chain (FTL) antibody (PA5-83567, Invitrogen), and ferritin heavy chain (FTH1) antibody (PA5-19058, Invitrogen), and with secondary antibodies, including Alexa Fluor Plus 405 Donkey Anti-Mouse IgG (A48257, Invitrogen), Alexa Fluor Plus 488 Donkey Anti-Rabbit IgG (A32790, Invitrogen), and Alexa Fluor Plus 555 Donkey Anti-Goat IgG (A48257, Invitrogen) as described in Supplementary Methods S1.4. Samples were analyzed under a Leica STELLARIS confocal microscope (Leica Microsystems, Wetzlar, Germany). Fluorescence signals for TFRC (blue), FTL (green), and FTH1 (yellow) were quantified using the ImageJ plugin Fiji (National Institutes of Health, Bethesda, MD, USA).^42^ Values were normalized to the red signal from propidium iodide (PI) to control for staining and imaging variability.

### Data Accessibility

Additional experimental data are provided in the Supplementary Materials. Unprocessed data are available in the Mendeley Data Repository (https://data.mendeley.com/datasets/n3gky25brh/1, doi:10.17632/n3gky25brh.1).

## Results

### Clinical Characteristics of Inter-Eye Asymmetry

To interrogate inter-eye asymmetry accompanying the KTCN, in the discovery study, 21 KTCN patients and six controls were recruited. More and less advanced eyes were classified based on ophthalmological examination according to the study workflow presented in Figure 1. Biological material was collected from both eyes during the CXL and PRK procedures for transcriptomic and proteomic profiling. Because of the doughnut pattern detectable in the CE of patients with KTCN, we implemented our previously designed protocol^7^ to define and separate three *topographic regions* embracing a thin cone center (*central topographic region*) surrounded by the thickened annulus (*middle topographic region*) and then a clinically unchanged peripheral area (*peripheral topographic region*). In total, six experimental samples were analyzed per individual.

**Figure 2.**
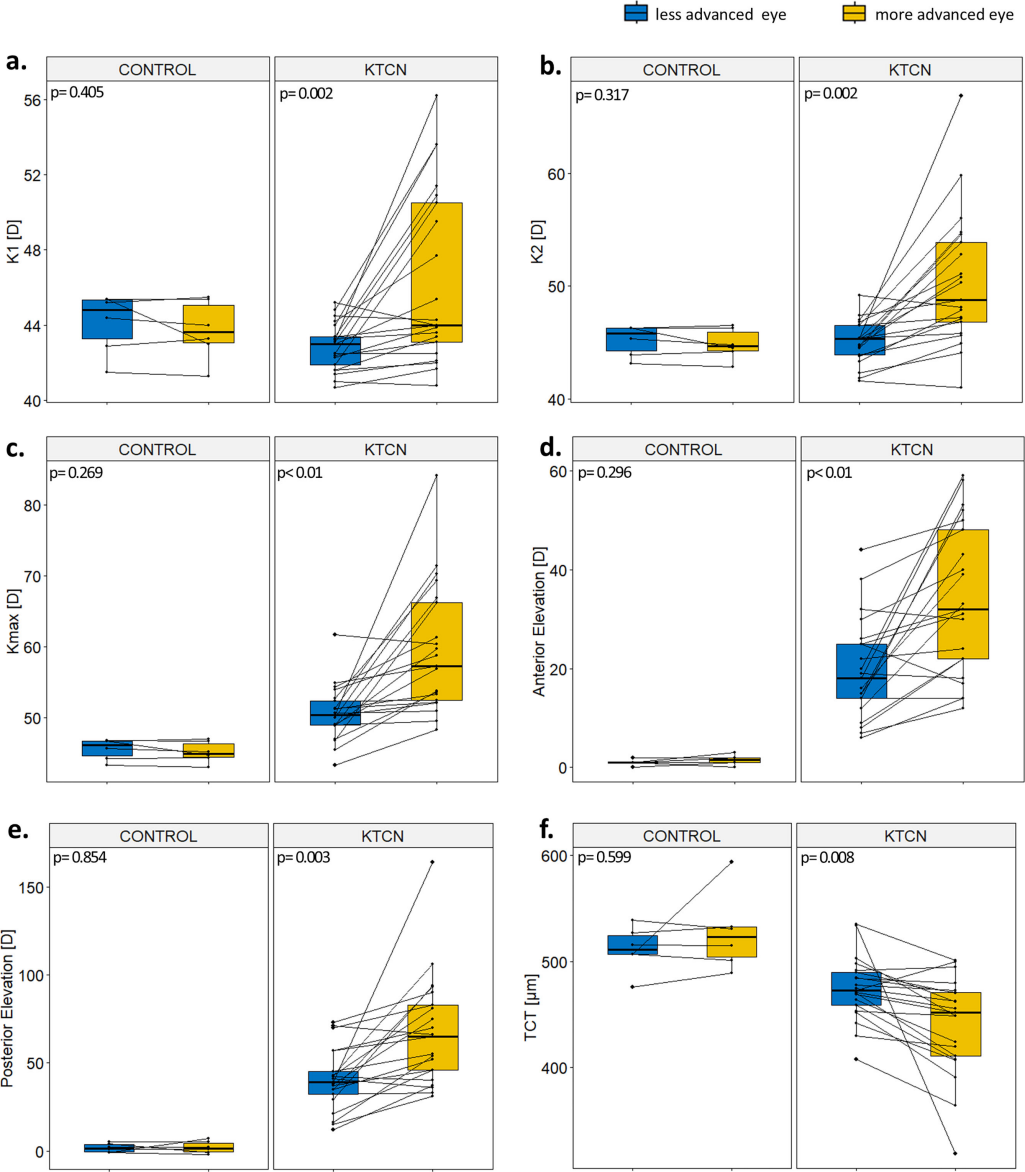
**Inter-eye asymmetry in clinical parameters in patients with KTCN.** Comparison of (**a**) flat keratometry (K1), (**b**) steep keratometry (K2), (**c**) maximal corneal curvature (K_max_), (**d**) anterior elevation, (**e**) posterior elevation, and (**f**) TCT values in more advanced (*yellow*) and less advanced (*blue*) eyes in patients with KTCN (*n =* 21 pairs) and mild myopia (as controls, *n =* 6). The *P* values of the two-tailed Wilcoxon signed-rank test are presented.

In patients with KTCN (*n* = 21), inter-eye asymmetry was observed in several clinical parameters, including lower TCT (*P* = 0.008) in more advanced eyes, alongside higher values of flat keratometry (K1; *P* = 0.002), steep keratometry (K2; *P* = 0.002), maximal corneal curvature (K_max_; *P* < 0.001), anterior elevation (*P* < 0.001), and posterior elevation (*P* = 0.003), as shown in Figure 2. The values of epithelial thickness in the *central topographic region* also were found to be different between pairs of KTCN eyes (*P* = 0.004). In contrast, in the control individuals (*n* = 6), no statistically significant differences in clinical parameters were found. The detailed clinical characteristics of both the patients with KTCN and control individuals are provided in Supplementary Table S2.

### Molecular Characteristics of Inter-Eye Asymmetry

#### Differentially Expressed Genes in a Paired Model of KTCN

**Figure 3.**
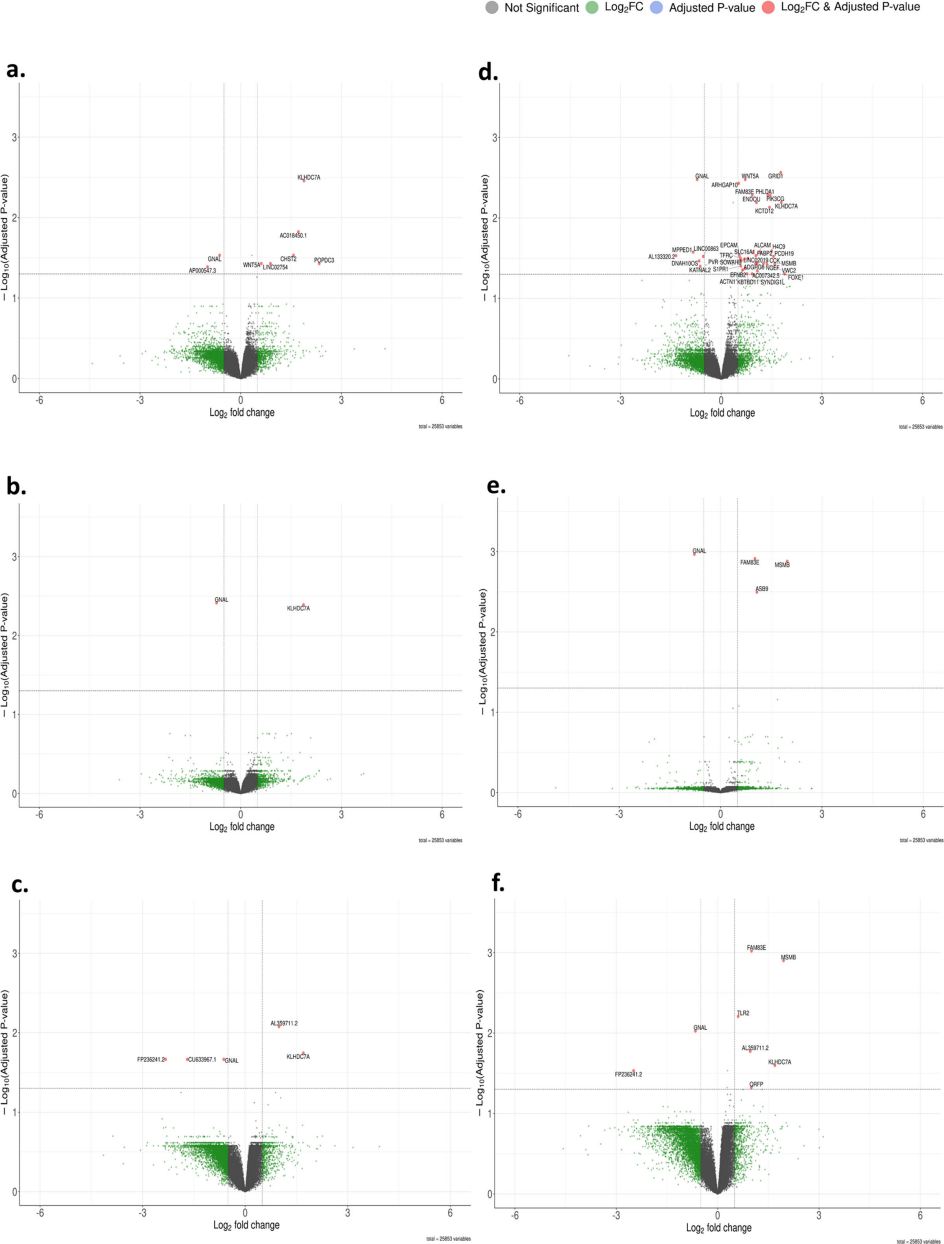
**Volcano plot of DEGs in the paired model of KTCN.** (**a**–**c**) Results of patients with KTCN whose inter-eye asymmetry expressed in the TKC grades was ≥1 (TKC difference ≥ 1; *n =* 6 pairs). A total of eight DEGs were identified for the *central topographic region* (**a**), two for the *middle topographic region* (**b**), and five for the *peripheral topographic region* (**c**). (**d**–**f**) Results of patients with KTCN whose inter-eye asymmetry expressed in the TKC grades was >1 (TKC difference >1; *n =* 5 pairs). A total of 37 DEGs were revealed for the *central topographic region* (**d**), four for the *middle topographic region* (**e**), and eight for the *peripheral topographic region* (**f**). The *green dots* represent genes fitting only the criterion of |log_2_FC| > 0.5, the *blue dots* indicate genes with downregulated expression (adjusted *P* < 0.05 and log_2_FC < −0.5), and the *red dots* denote upregulated gene expression (adjusted *P* < 0.05 and log_2_FC > 0.5). See Supplementary Table S4 for more details.

The transcriptomic profiles were established based on 96 and 36 experimental samples derived from 16 patients with KTCN and six controls, respectively (Supplementary Table S3). To evaluate whether clinical inter-eye asymmetry is mirrored at the transcriptomic level, we performed series of comparisons of the paired eyes, progressively restricting the cohort to subsets with increasing inter-eye clinical disparity (all patients; patients with a TKC inter-eye difference ≥ 1; and patients with TKC difference > 1; see Materials and Methods). In the analysis embracing all recruited patients with KTCN, no differentially expressed genes (DEGs) were found (volcano plots for the *central*, *middle*, and *peripheral topographic regions* are shown in Supplementary Figs. S1a–S1c). Restricting the analysis to patients with greater interocular clinical asymmetry statistically significant results were obtained. Paired comparisons in patients with a TKC inter-eye difference ≥ 1 are presented in Figures 3a to 3c, and results for the patient subgroup with TKC difference > 1 are shown in Figures 3d to 3f. The revealed 48 DEGs are presented in Table 1, with detailed data provided in Supplementary Table S4. To assess physiological inter-eye variability and validate disease specificity, the eyes from controls were evaluated. No DEGs were discovered in pairs of CE samples of controls (Supplementary Figs. S1d–S1f).

**Table 1.** DEGs in Paired Model of KTCN

Analysis Input	Gene	Log_2_FC	Adjusted *P*
*Central topographic region* of KTCN patients with TKC difference ≥ 1	*KLHDC7A*	1.8823	0.0035
	*AC018450.1*	1.7176	0.0150
	*CHST2*	1.5704	0.0294
	*GNAL*	−0.6274	0.0294
	*LINC02754*	0.8848	0.0372
	*POPDC3*	2.3370	0.0372
	*WNT5A*	0.6143	0.0372
	*AP000547.3*	−0.9888	0.0410
*Central topographic region* of KTCN patients with TKC difference > 1	*GRID1*	1.7750	0.0027
	*GNAL*	−0.7168	0.0033
	*WNT5A*	0.7156	0.0033
	*ARHGAP10*	0.5229	0.0037
	*FAM83E*	0.9278	0.0052
	*PHLDA1*	1.3883	0.0052
	*PIK3CG*	1.4599	0.0052
	*ENDOU*	1.0504	0.0064
	*KLHDC7A*	1.7899	0.0064
	*KCTD12*	1.4392	0.0074
	*H4C9*	1.5024	0.0258
	*FABP2*	1.1698	0.0266
	*MPPED1*	−0.8337	0.0266
	*SLC16A1*	0.9422	0.0269
	*AL133320.2*	−1.3465	0.0294
	*ALCAM*	1.0308	0.0294
	*EPCAM*	0.5411	0.0294
	*LINC00863*	−0.5343	0.0301
	*PCDH19*	1.5566	0.0301
	*LINC02019*	0.7978	0.0318
	*TFRC*	0.5798	0.0318
	*PVR*	0.7043	0.0325
	*SOWAHB*	0.5977	0.0341
	*DNAH10OS*	−0.6567	0.0341
	*ADGRG6*	1.0219	0.0368
	*CCK*	1.2559	0.0368
	*NGEF*	1.0702	0.0368
	*MSMB*	1.5936	0.0375
	*VWC2*	1.3635	0.0375
	*S1PR1*	0.5862	0.0395
	*KATNAL2*	−0.6232	0.0399
	*EFNB2*	0.7208	0.0424
	*ACTN1*	0.6381	0.0441
	*AC007342.5*	1.0750	0.0454
	*KBTBD11*	0.7690	0.0494
	*SYNDIG1L*	0.9225	0.0494
	*FOXE1*	1.8865	0.0494
*Middle topographic region* of KTCN patients with TKC difference ≥ 1	*GNAL*	−0.7215	0.0039
	*KLHDC7A*	1.8706	0.0041
*Middle topographic region* of KTCN patients with TKC difference > 1	*GNAL*	−0.7759	0.0011
	*FAM83E*	1.0172	0.0012
	*MSMB*	1.9729	0.0013
	*ASB9*	1.0734	0.0032
*Peripheral topographic region* of KTCN patients with TKC difference ≥ 1	*AL359711.2*	0.9905	0.0084
	*KLHDC7A*	1.7031	0.0180
	*CU633967.1*	−1.6939	0.0216
	*FP236241.2*	−2.3280	0.0216
	*GNAL*	−0.6263	0.0216
*Peripheral topographic region* of KTCN patients with TKC difference >1	*FAM83E*	1.0036	0.0010
	*MSMB*	1.9496	0.0013
	*TLR2*	0.6073	0.0062
	*GNAL*	−0.6579	0.0094
	*AL359711.2*	0.9651	0.0168
	*KLHDC7A*	1.6927	0.0252
	*FP236241.2*	−2.4865	0.0293
	*QRFP*	0.9985	0.0477

Results of analysis in two setting are presented: (1) patients with KTCN whose inter-eye asymmetry expressed in the TKC grades was ≥1 (TKC difference ≥ 1; *n* = 6 pairs), and (2) patients with KTCN whose inter-eye asymmetry expressed in the TKC grades was >1 (TKC difference > 1; *n* = 5 pairs). For each *topographic region* (*central*, *middle*, or *peripheral*) and each setting, the analysis was performed separately; the presented *P* values were adjusted for multiple comparisons using the Benjamini–Hochberg correction method in the limma package.^34^^,^^35^ See Supplementary Table S4 for more details.

#### Molecular Features of Disease Status and Severity in KTCN

**Figure 4.**
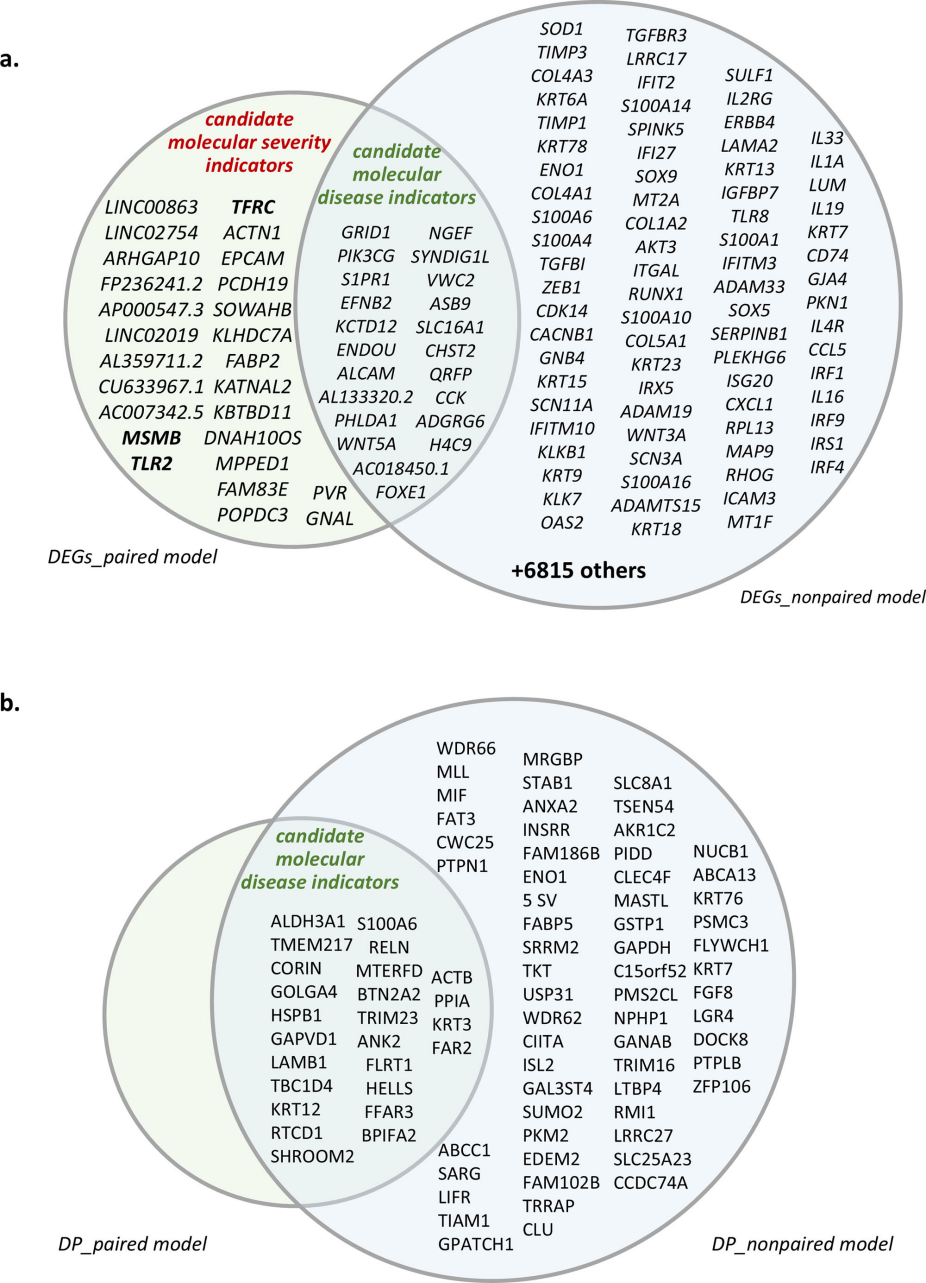
**Venn diagram of DEGs and discriminative proteins in the paired and non-paired model in the paired and non-paired model.** (**a**) Differential expression analysis was conducted under two analytical frameworks: paired and non-paired models, using the limma package.^34^^,^^35^ In the paired model, more and less affected KTCN eyes were assigned in each eye pair, and differential analysis within pairs of CE samples was then performed in two settings: (1) patients with KTCN whose inter-eye asymmetry expressed in the TKC grades was ≥1 (TKC difference ≥ 1; *n =* 6 pairs), and (2) patients with KTCN whose inter-eye asymmetry expressed in the TKC grades was >1 (TKC difference > 1; *n =* 5 pairs). In the non-paired model, all KTCN samples were compared to all control samples (KTCN group, *n =* 50 vs. control group, *n =* 14). The Venn diagram illustrates the overlap and divergence between DEGs identified in the two models. Non-overlapping portions of the circles (*green* and *blue*) indicate DEGs unique to each approach. The overlapping area represents shared DEGs that may serve as molecular disease indicators; the 26 genes shown in the *green* non-overlapping portion of the circle constitute candidates for molecular indicators of KTCN severity. Genes selected for further analysis are shown in *bold*. (**b**) Analysis of discriminative proteins of CE was performed under two analytical frameworks: paired and non-paired models. In the paired model, more and less affected KTCN eyes were assigned in each eye pair, and differential analysis within pairs of CE samples was then performed in three settings: (1) all patients with KTCN (*n =* 36 pairs), (2) patients with KTCN whose inter-eye asymmetry expressed in the TKC grades was ≥1 (TKC difference ≥ 1; *n =* 8 pairs), and (3) patients with KTCN whose inter-eye asymmetry expressed in the TKC grades was >1 (TKC difference >1; *n =* 7 pairs). In the non-paired model (for KTCN group *n =* 42, for control group *n =* 14) analysis embraced all KTCN samples versus all control samples. The similarities between both analyses (paired and non-paired models) are presented in the overlapping portion of the circles. All 25 proteins identified as exhibiting molecular inter-eye asymmetry in KTCN were also found to be significant in the analysis using the non-paired model (KTCN vs. controls).

After the DEG paired analysis was completed, another DEG analysis, embracing all currently and previously generated RNA-sequencing (RNA-seq) data^7^ was performed (in a non-paired analysis model) to identify transcriptomic alterations, within the KTCN cohort, associated with advanced disease. In this analysis, 3816 (857 protein-coding) DEGs for the *central topographic region*, 2440 (546 protein-coding) DEGs for the *middle topographic region*, and 1194 (466 protein-coding) DEGs for the *peripheral topographic region* were found. Then, overlapping DEGs in the paired and unpaired assessments were excluded from the paired analysis model results, as the differential expression of these removed DEGs could be an effect of the disease status (possible molecular disease indicators are indicated in Fig. 4a). As a final effect, 26 (17 protein-coding) candidates for molecular indicators of KTCN severity were revealed (Fig. 4a). Overrepresentation analysis (ORA) of this set of genes indicated enrichment of one GO-based term—namely cell–cell adhesion (GO category: Biological Processes, GO ID: 0098609) (adjusted *P* = 0.027), with *ACTN1*, *EPCAM*, *PCDH19*, *PVR*, and *TFRC* genes contributing to this process.

#### Proteomic Profiles

Proteomic profiles were obtained based on 126 experimental samples derived from 21 patients with KTCN and 36 experimental samples from six control individuals involved in the discovery study, and they were analyzed in the paired model to evaluate the extent to which the magnitude of clinical inter-eye asymmetry is reflected in proteomic variability. Fragments of 25 unique proteins were revealed as discriminative between eyes in patients with KTCN, including 11, 4, and 15 proteins for *central*, *middle*, and *peripheral topographic regions*, respectively (*P* < 0.01) (Table 2, Supplementary Table S5).

**Table 2.** Discriminative Proteins in the Paired Model of KTCN

	*Topographic Region*		
Analysis Input	*Central*	*Middle*	*Peripheral*
All KTCN patients (*n* = 36)	TRIM23, HSPB1, RTCD1, KRT3, FAR2, KRT12, CORIN, PPIA, LAMB1, BPIFA2, ACTB	SHROOM2, TBC1D4, S100A6	FFAR3, TMEM217, ALDH3A1, PPIA, HSPB1, MTERFD2, KRT3, HELLS, GAPVD1, FLRT1, KRT12, CORIN
KTCN patients with TKC difference ≥ 1	—	—	HSPB1, FLRT1, ANK2, BTN2A2
KTCN patients with TKC difference > 1	—	RELN	HSPB1, ANK2, KRT12, GOLGA4

Results of analysis in three settings are presented: (1) all patients with KTCN (*n =* 36 pairs), (2) patients with KTCN whose inter-eye asymmetry expressed in the TKC grades was ≥1 (TKC difference ≥ 1; *n =* 8 pairs), and (3) patients with KTCN whose inter-eye asymmetry expressed in the TKC grades was >1 (TKC difference > 1; *n* = 7 pairs). For each *topographic region* (*central*, *middle*, or *peripheral*) and each setting, analysis was performed separately. All *m*/*z* values, fragment sequence, protein name, and *P* values of the Mann–Whitney test are presented in Supplementary Table S5.

**Figure 5.**
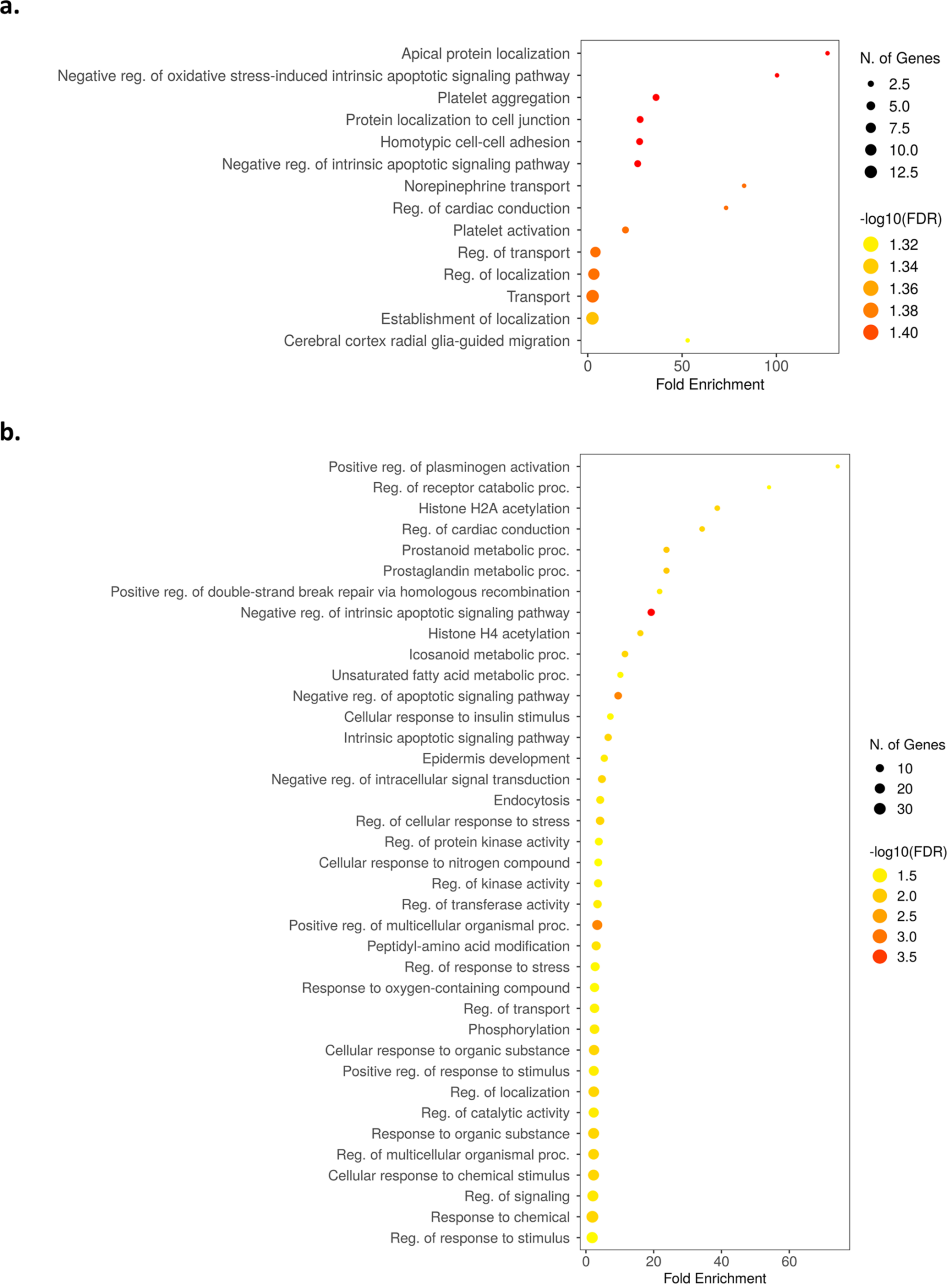
**Results of ORA of GO terms in the set of discriminative proteins.** (**a**, **b**) ORA analysis was performed and visualized for a category of Biological Processes in the paired model (**a**) and non-paired model (**b**) using ShinyGO.^41^ In the paired model, more and less affected KTCN eyes were assigned in each eye pair, and differential analysis within pairs of CE samples was then performed in three settings: (1) all patients with KTCN (*n =* 36 pairs), (2) patients with KTCN whose inter-eye asymmetry expressed in the TKC grades was ≥1 (TKC difference ≥ 1; *n =* 8 pairs), and (3) patients with KTCN whose inter-eye asymmetry expressed in the TKC grades was >1 (TKC difference > 1; *n =* 7 pairs). In the non-paired model (for KTCN group *n =* 42, for control group *n =* 14), the analysis embraced all KTCN samples versus all control samples (**b**). GO terms are listed on the *y*-axis, and the *x*-axis represents fold enrichment, which is defined as the percentage of genes in a given list belonging to a pathway divided by the corresponding percentage in the background (all protein-coding genes in the genome). The *size of the dot* represents the number of discriminative proteins in the particular GO term set. The *color scale* indicates the –log_10_ transformed FDR; the lowest FDR values are indicated in *red*. *P* ≤ 0.05 and/or FDR ≤ 0.05 were the cutoff values.

The ORA analysis of this set of proteins indicated overrepresentation of 10 GO-based terms in the category of Biological Processes, including apical protein localization (GO ID: 0045176) (FDR < 0.05), with ACTB and SHROOM2 involved; protein localization to cell junction (GO ID: 1902414) (FDR < 0.05), with ACTB, RELN, and HSPB1 contributing; and homotypic cell–cell adhesion (GO ID: 0034109) (FDR < 0.05), with ACTB, HSPB1, and PPIA playing a role (Fig. 5a, Supplementary Table S6).

Then, to further distinguish molecular features related to disease status, an analysis of discriminative proteins was performed in the non-paired model that encompassed all currently and previously generated MALDI-MS data derived from KTCN versus control corneal material.^7^ In this analysis, fragments of 86 proteins were identified as specific for KTCN disease status (Supplementary Table S7). In the ORA analysis of this protein set, the overrepresentation of 38 GO-based terms in the category of Biological Processes (Fig. 5b, Supplementary Table S8) was identified. Notably, these processes included apoptosis, responses to various stimuli, and histone-related functions. Finally, the above-mentioned set of 86 proteins was used to point to proteins solely attributable to disease severity, following the analysis workflow described for DEGs. None of the proteins identified in the paired-samples analysis was unique (Fig. 4b).

#### Rediscovery Study and Components of the KTCN Severity Signature in the Context of Disease Pathophysiology

Potential components of the KTCN severity signature were verified/validated in a rediscovery study involving an additional 20 patients with KCTN undergoing the CXL procedure (details of clinical characteristics of these patients are presented in Supplementary Table S9). In this cohort, the clinical inter-eye asymmetry was examined and confirmed based on several clinical parameters. More advanced eyes exhibited significantly lower TCT values (*P* < 0.001) and higher values of K1 (*P* < 0.001), K2 (*P* < 0.001), K_max_ (*P* < 0.001), anterior elevation (*P* < 0.001), and posterior elevation (*P* < 0.001), as shown in Supplementary Figure S2.

From an initial set of 26 candidates (Fig. 4a), the *TFRC*, *MSMB*, and *TLR2* genes were selected for further assessment as potential molecular indicators of disease severity based on their involvement in particular biological processes and the correlation between their expression and clinical data in the discovery study (Supplementary Fig. S3). According to the results of DEG analysis in the paired model, the relative expression of the *TFRC* gene was analyzed in CE samples of all three *topographic regions*, the *MSMB* expression was validated for CE samples of the *middle topographic region*, and, in the case of *TLR2*, expression was examined for CE samples of the *peripheral topographic region*. In the rediscovery study, the higher relative expression of *TFRC* was confirmed in the *middle topographic region* of eyes with more advanced disease compared to their less affected counterparts (*P* = 0.008, paired samples *t*-test) (Supplementary Fig. S4a, Supplementary Table S10). As found in the RNA-seq data, no significant differences in *TFRC* expression were identified in the *peripheral topographic region* (Supplementary Fig. S4a). The change in expression of *MSMB* and *TLR2* genes between more and less advanced eyes was not confirmed in the rediscovery group using RT-qPCR (Supplementary Figs. S4b, S4c; Supplementary Table S10).

To further examine the biological significance of results, the correlations between the expression of selected genes and clinical data in the rediscovery study were evaluated. *TFRC* expression in the *middle topographic region* of the CE was found to correlate with average *topographic region* thickness (Spearman correlation coefficient *r* = 0.53, *P* = 0.008) and K1 (*r* = 0.49, *P* = 0.016) (Supplementary Fig. S5). No correlation was found between the expression of *MSMB* and *TLR2* genes and clinical parameters (Supplementary Fig. S6).

**Figure 6.**
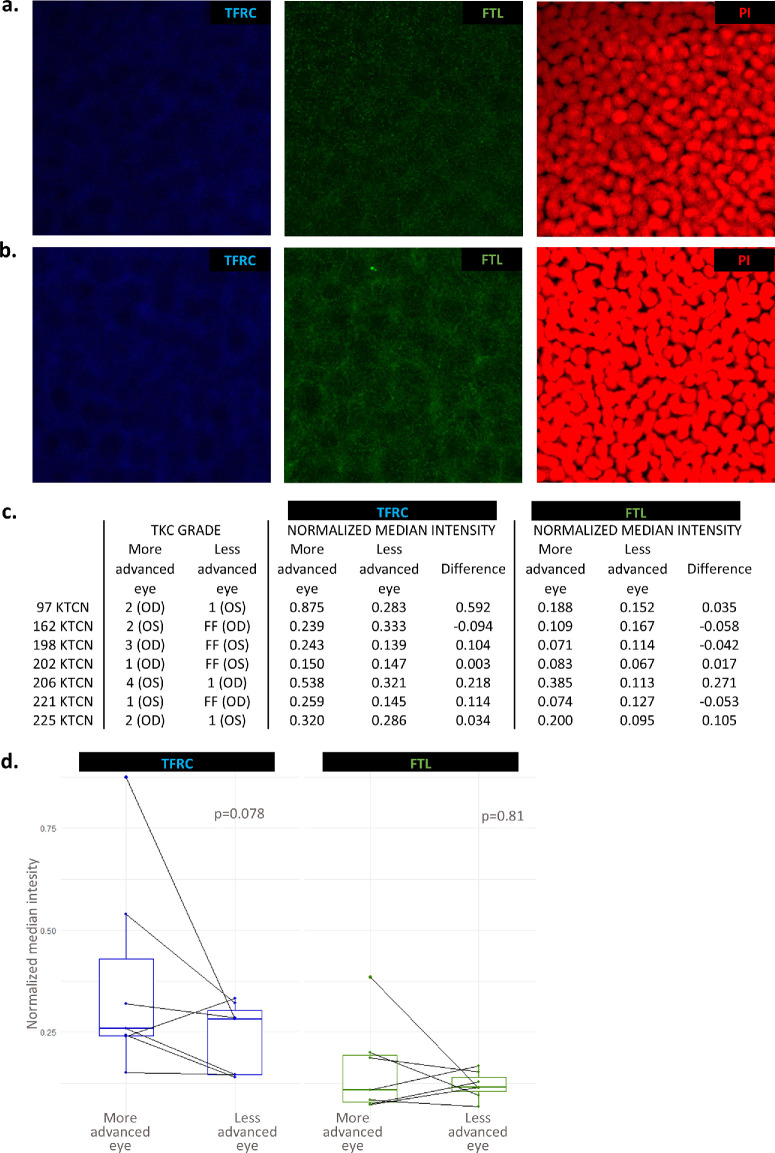
**The representative expression profile of TFRC (*blue*) and FTL (*green*) proteins in CE samples of a patient with KTCN.** (**a**, **b**) Immunofluorescence staining of CE samples from the *middle topographic region* of a KTCN patient (#221 KTCN), showing the eye with less advanced disease (#221 KTCN/OD, forme fruste KTCN) (**a**) and the eye with more advanced disease (#221 KTCN/OS) (**b**). (**c**) Quantitative analysis of TFRC and FTL expression in the *middle topographic region* of CE samples from seven KTCN patients. Fluorescence intensities were quantified using the ImageJ plugin Fiji,^42^ based on median fluorescence values normalized to nuclear signal obtained from propidium iodide (PI) staining (*red*). The TKC grade is shown for both the less and more affected eyes of each patient. Normalized median fluorescence values and intra-patient differences are presented. (**d**) Paired data visualization of TFRC and FTL immunofluorescence staining, including *P* values from paired samples *t*-tests. Normalized median fluorescence values are shown on the *y*-axis, and the *x*-axis indicates the more and less advanced eyes. *Z*-stack images were acquired using a Leica STELLARIS confocal microscope (HC PL APO CS2 20×/0.75 DRY objective, digital zoom 2.0, and the “max” intensity projection mode in LAS X 4.6.1 software). Detailed imaging parameters are available in the metadata file hosted on the Mendeley Data Repository (https://data.mendeley.com/datasets/n3gky25brh/1; doi:10.17632/3nxdd8kc4v.1). See Supplementary Table S9 for clinical details and Supplementary Table S11 for IF staining quantification. FF, forme fruste; OS, oculus sinister (left eye); OD, oculus dexter (right eye).

**Figure 7.**
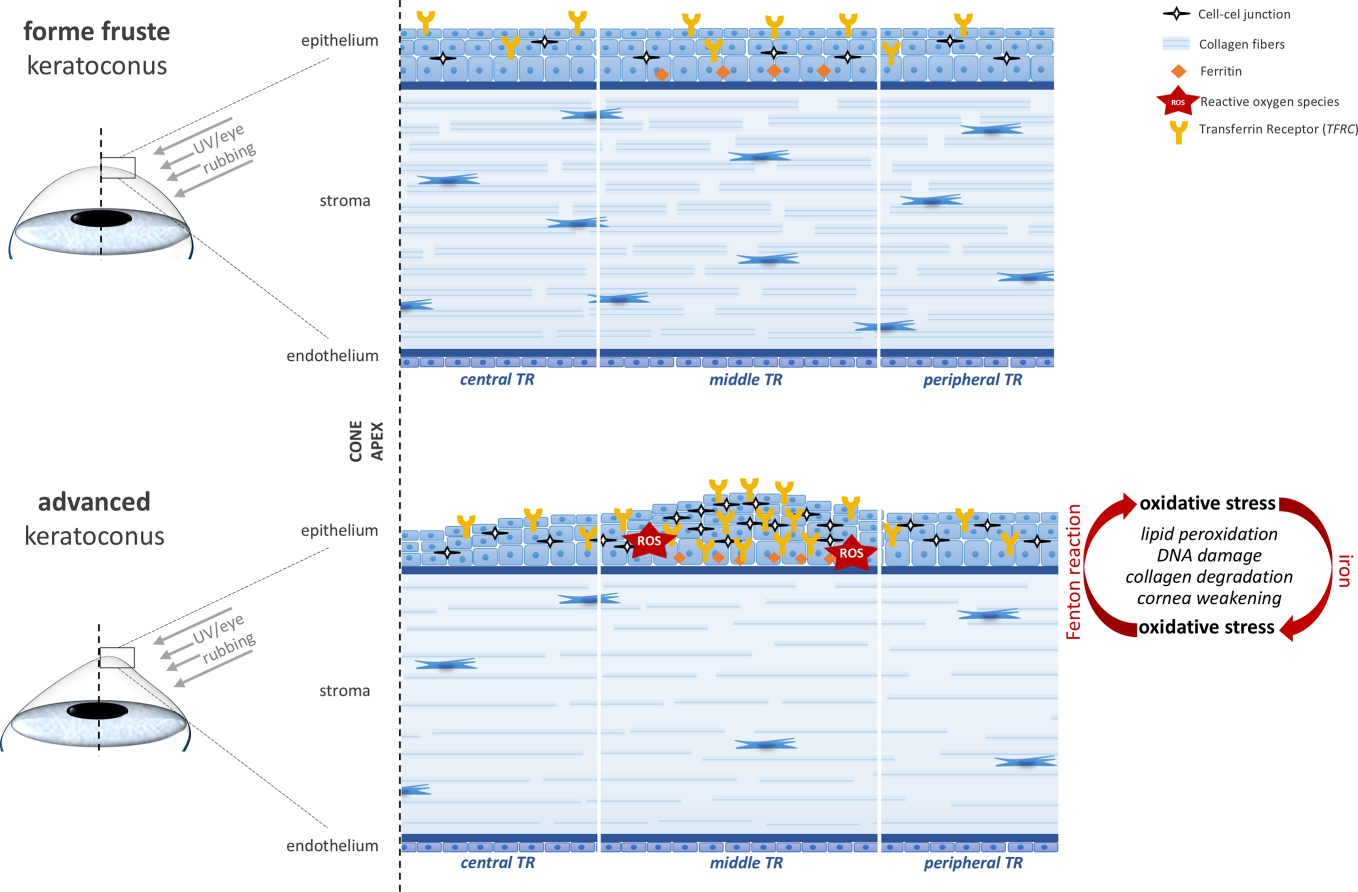
**Hypothetical mechanism underlying KTCN advancement.** This model, summarizing components of the KTCN molecular signature, was developed based on findings from the rediscovery study and previous research.^7^^,^^18^^,^^43^^,^^44^ The mechanism highlights the generation of reactive oxygen species formed as a result of both exogenous factors, such as ultraviolet light exposure and mechanical trauma from eye rubbing, and endogenous factors, notably increased iron uptake via TFRC. Excess intracellular iron, when not properly sequestered, catalyzes the Fenton reaction, further amplifying reactive oxygen species production and promoting oxidative stress. These processes contribute to lipid peroxidation, damage to cellular membranes and DNA, collagen degradation, and, ultimately, biomechanical weakening of the cornea. In parallel, CE cells exposed to oxidative stress may present elevated expression of adhesion-related genes as a compensatory response to preserve epithelial integrity, with changes varying for each *topographic region* (*central*, *middle*, or *peripheral*). Together, these factors contribute to oxidative stress–induced CE remodeling and the underlying pathophysiological mechanisms of KTCN advancement.

Next, to determine whether transcriptional alterations of *TFRC* are reflected at the protein level, immunofluorescence (IF) staining of CE samples from seven KTCN patients was performed. The analysis confirmed both intra-individual and region-specific differences in TFRC protein expression. Notably, TFRC expression was elevated in the *middle topographic regions* of the more advanced eyes compared to the less affected eyes, including those with forme fruste KTCN (Fig. 6, Supplementary Table S11). In parallel, ferritin light- and heavy-chain staining was assessed to evaluate whether increased *TFRC* expression translated into altered iron storage. In contrast to TFRC staining, no significant intra-individual and/or *topographic region*-specific differences were observed (Fig. 6, Supplementary Table S11). Finally, integrating the results of the rediscovery study with our prior findings,^7^^,^^18^^,^^43^^,^^44^ we propose a mechanism underlying KTCN advancement, integrating oxidative stress with endogenous and exogenous determinants implicated in the disease pathophysiology, as illustrated in Figure 7.

## Discussion

The pathophysiology of KTCN involves numerous molecular and cellular changes that contribute to degradation of the corneal structure. In this context, alterations in the CE, evaluated in detail in this study, including previously reported variations in cell adhesion and the imbalance of oxidative stress,^7^^,^^17^^,^^21^^,^^22^ could constitute molecular components of the signatures associated with disease severity and progression.

In this study, the cell–cell adhesion-related processes were indicated as the most specific pathway in determining the severity of the KTCN, considering both transcriptomic and proteomic CE profiles (results of analyses in the paired model). These processes in the cornea are essential for maintaining the structural integrity and function of the tissue.^45^^–^^47^ The corneal epithelium is a dynamic cellular layer that forms the first line of defense against environmental factors/stressors, while also playing a critical role in maintaining the transparency and mechanical properties of the cornea. The coordination of these concurrently occurring processes relies on the proper function of cell junctions, including tight junctions, adherent junctions, and desmosomes, which facilitate cellular cohesion and communication between epithelial cells and underlying stromal layers.^48^ In KTCN, alterations in these adhesion mechanisms have been widely reported. Studies have demonstrated a reduced expression of key adhesion molecules, such as E-cadherin, which plays a critical role in epithelial cell–cell interactions.^19^ The loss of E-cadherin integrity in KTCN patients has been linked to disruption of the epithelial barrier function, allowing environmental factors to further destabilize the corneal structure.^49^ Furthermore, alterations in integrin expression, particularly with respect to *ITGB1*, which mediate the attachment of epithelial cells to the basement membrane have been observed in KTCN.^7^ Also, in a multi-ethnic genome-wide association study of KTCN, a significant association near the integrin gene *ITGA2* (coding the complexes of integrin α2β1) was found.^50^ We previously reported the overlap between transcriptomic and genomic findings embracing the pathway of integrin-mediated adhesion of the cells to the extracellular matrix.^7^^,^^44^ Such disruptions compromise the corneal epithelial integrity, contributing to an impaired attachment of the epithelium to the underlying stroma, which could exacerbate the thinning and biomechanical weakening characteristic of KTCN.

Here, we identified *TFRC* as the most specific indicator, which correlates with the severity of KTCN, suggesting that *TFRC* may be a significant component of the KTCN severity signature. Previously, studies have shown that transferrin receptor expression increases in response to cellular stress, including reactive oxygen species such as H_2_O_2_, as shown in murine fibroblasts.^51^ Oxidative stress is a well-recognized factor in the pathophysiology of KTCN^21^^,^^52^^,^^53^ and can be induced by exogenous factors (e.g., ultraviolet light exposure, mechanical trauma from eye rubbing),^54^ as well as endogenous disturbances (e.g., mitochondrial dysfunction caused by telomere shortening,^55^ a hypersensitive oxidative response,^56^ and higher burden of non-synonymous variants in mitochondrial complex I genes^57^). Resulting reactive oxygen species can lead to lipid peroxidation, DNA damage, and the degradation of collagen fibers, which, together with other stochastic molecular changes, contribute to the progressive weakening of both corneal stroma and CE in KTNC.^58^ Moreover, other studies have shown that elevated levels of *TFRC* expression in CE cells may be indicative of increased iron uptake, which is likely to lead to iron accumulation in the tissue.^59^ The accumulation of iron within the corneal tissue is observed during the course of the disease in the form of the Fleischer ring,^60^ although its occurrence has been associated with disease duration rather than severity.^61^ Thus, increased TFRC may reflect an early, stress-related molecular response that precedes the detectable iron deposition. The former is consistent with our finding of no differences found in stored iron levels, as indicated by ferritin light (FTL) and heavy (FTH1) chain staining. Although iron plays a crucial role in several biological processes, including cellular metabolism, DNA synthesis, and oxygen transport, dysregulated iron levels could contribute to oxidative stress and tissue damage. Recent research has shown that ferritin can also be present in the nucleus of corneal epithelial cells, where it may play an important antioxidant role. Cai et al.^62^ proposed that nuclear ferritin may help protect the corneal DNA from oxidative damage, but the interplay between oxidative stress and iron metabolism is complex and bidirectional, as the excess iron can catalyze the formation of hydroxyl radicals through the Fenton reaction, further amplifying oxidative stress. Moreover, iron itself may directly contribute to thinning of the corneal stroma, as it is required for the formation of hydroxylysine, an amino acid involved in collagen cross-linking; therefore, it is essential for the synthesis of collagen fibers.^63^^,^^64^

Although *TLR2* and *MSMB* were identified as promising molecular indicators of the KTCN severity signature in RNA-seq data (the discovery study), we were unable to confirm their association with disease severity in the rediscovery group. *TLR2* is involved in the innate immune response and has been implicated in inflammation and tissue remodeling,^65^ whereas *MSMB* (mesothelin) is a cell surface glycoprotein that may play a role in cell adhesion and proliferation.^66^ Despite their promising (in the context of CE physiology/pathology) molecular functions, these genes did not show a consistent correlation with KTCN severity in our rediscovery cohort, suggesting that further research is necessary to fully understand their potential role in KTCN.

Despite the substantial findings of this study, several limitations should be acknowledged. First, our sample size for a paired-model analysis (21 pairs of eyes with KTCN) was sufficient in terms of statistical significance in the analyses but could be expanded in future studies. To strengthen our conclusions, our key observations were supported by results from a larger non-paired cohort (KTCN group, *n* = 42; control group, *n* = 14) and further validated in a rediscovery group (*n* = 20). Additional studies involving diverse patient populations and incorporating longitudinal sampling (for example, through impression cytology) are necessary to confirm the link between *TFRC* expression and disease severity and progression. Second, although we identified a strong correlation between *TFRC* expression and KTCN severity in the corneal epithelium, we did not investigate the broader molecular landscape of oxidative stress and iron metabolism.

In conclusion, in the implemented paired model of analyses, the significance of the results shifted toward findings more specifically associated with disease severity. We highlight that *TFRC*, in combination with clinical data, may offer a valuable prognostic tool for assessing disease advancement in KTCN. The interplay between oxidative stress and iron dysregulation, described components of the KTCN severity signature, forms the vicious cycle that accelerates corneal degeneration in KTCN. These findings underscore the need for further investigation into whether these processes are involved in the disease pathophysiology. Moreover, identification of molecular features that precede the iron accumulation is not only of scientific interest, as it may help clarify the underlying mechanisms of the disease, but also of clinical relevance. These findings open the door to potential therapeutic strategies aimed at targeting oxidative stress and iron metabolism to prevent or slow the progression of KTCN.

## Supplementary Material

Supplement 1
