# Supplementary material for: Inter-Eye Molecular Discrepancies in the Corneal Epithelium Point to TFRC in the Keratoconus Severity Signature and Mechanism of Cone Formation
Source: Invest Ophthalmol Vis Sci. 2025 Oct 29;66(13):52. doi: 10.1167/iovs.66.13.52 (PMC12577770; doi:10.1167/iovs.66.13.52)
Supplement: Supplement 1 [file iovs-66-13-52_s001.pdf]

For Investigative Ophthalmology & Visual Science

SUPPLEMENTARY MATERIALS

Inter-eye Molecular Discrepancies in the Corneal Epithelium Point to *TFRC* in the Keratoconus Severity Signature and Mechanism of Cone Formation

Katarzyna Jaskiewicz-Rajewicz[1], Alicja Wysocka[1], Magdalena Maleszka-Kurpiel[2,3], Eliza Matuszewska-Mach[4], Jakub Wozniak[5,6], Rafal Ploski[7], Jan Matysiak[4], Malgorzata Rydzanicz[7], Marzena Gajecka[1, 5]

[1] Institute of Human Genetics, Polish Academy of Sciences, Poznan, Poland
[2] Optegra Eye Health Care Clinic in Poznan, Poland
[3] Poznan University of Medical Sciences, Chair of Ophthalmology and Optometry, Poznan, Poland
[4] Poznan University of Medical Sciences, Chair and Department of Inorganic and Analytical Chemistry, Poznan, Poland
[5] Poznan University of Medical Sciences, Chair and Department of Genetics and Pharmaceutical Microbiology, Poznan, Poland
[6] Department of Genetics and Animal Breeding, Poznan University of Life Sciences, Poznan, Poland
[7] Department of Medical Genetics, Medical University of Warsaw, Warsaw, Poland

1. SUPPLEMENTARY METHODS

1. 1. Patients and control individuals, clinical evaluation

The JASP Software(JASP Team, 2022) was used in statistical evaluation of clinical features and parameters as described in Supplementary Materials 1.1. The normality of continuous data was assessed by the Shapiro-Wilk test. The paired t-test was applied for continuous variables with a normal distribution. If the normality assumption was not satisfied, the Wilcoxon signed-rank test was conducted. In case of more than two-group analyses, the Repeated Measures ANOVA was executed. The post-hoc testing was performed using the Holm-Bonferroni method to correct for multiple testing. For all performed statistical tests p-values <0.05 were considered statistically significant. The study was designed as a retrospective case-control study, therefore, sample size analysis with a priori power analysis was not performed; instead, effect size metrics/indices were verified for all statistically significant results concerning clinical parameters (Cohen's d > 0.5).

1.2. MALDI-TOF/TOF MS/MS Protein-Peptide Profiling

During MALDI-TOF/TOF MS/MS proteomic analysis, each experimental sample was manually spotted onto the MTP AnchorChip 800 μm target plate (Bruker Daltonics, Bremen, Germany) in three technical replicates. The means of these three technical replicates were then analyzed in subsequent profiling. Peaks (representing different protein/peptide fragments) with missing values in more than 30% of all samples were excluded from the analysis, and the remaining missing values were filled with half of the minimum value. In the final statistical analysis, conducted using JASP Software(JASP Team, 2022), 405 out of 1,484 peaks were included. The data did not meet the assumption of normality (as confirmed by the Shapiro-Wilk test), so the paired-samples Wilcoxon signed-rank test was applied. All p-values < 0.01 were considered statistically significant (designated as discriminative peaks). Only these discriminative peaks were selected for further identification using the SwissProt protein

sequence database. Not all discriminative peaks were unambiguously identified in the SwissProt database.

1.3. RNA-Seq data validation in the rediscovery study using RT-qPCR

To validate the study results, additional pairs of CE samples were collected and processed according to the study workflow (Figure 1), followed by RT-qPCR reactions. Briefly, RNA samples from the rediscovery study were reverse transcribed to cDNA using the Maxima First Strand cDNA Synthesis Kit for RT-qPCR with dsDNase (Thermo Fisher Scientific Inc., Lithuania), following the manufacturer's instructions. Expression levels of selected genes were quantified using HOT FIREPol EvaGreen qPCR Mix Plus (Solis BioDyne OÜ, Estonia) and the CFX96 Touch Real-Time PCR Detection System (Bio-Rad Laboratories, Hercules, CA, USA), according to the manufacturer's protocol. Each reaction was performed in triplicates, and if one replicate deviated by ≥0.5 Ct, it was excluded, and two replicates were used in subsequent calculations. Primer sequences and annealing temperatures are listed in Supplementary Table S1. Relative gene expression levels were normalized to *UBC*, *LDHA*, and *RPL4* transcript levels using the comparative CT method. Reference genes were selected based on previous studies and expression data from our KTCN research. The Mann-Whitney U test was used to assess differences in gene expression levels within pairs of samples and compared to a whole set (based on ΔΔCt values), and box plots were generated to visualize the results.

1.4. Immunofluorescence (IF) staining of CE samples

The CE samples were rinsed 3x with PBS (pH 7.4) and then fixed for 10 min in ice-cold methanol at -20°C. After incubation, samples were rinsed with PBS (3x) followed by adding a blocking buffer containing 5% BSA, 0,3% Triton, and 10% normal donkey serum (ab7475, Abcam) in PBS (pH 7.4) for 1 hour at room temperature. Subsequently, the samples were incubated overnight at 4°C with primary TFRC antibody (#13-6800, Invitrogen), primary FTL antibody (#PA5-83567, Invitrogen), and primary FTH1 antibody (PA5-19058, Invitrogen) at

concentrations 1:150, 1:150, and 1:100, respectively. Next, the tissues were rinsed three times with PBST (PBS with 0.3% Triton) and incubated for 2 hours at room temperature in the dark with secondary antibodies Alexa Fluor® Plus 405 donkey anti-mouse IgG (A48257, Invitrogen), Alexa Fluor® Plus 488 donkey anti-rabbit IgG (A32790, Invitrogen), and Alexa Fluor® Plus 555 donkey anti-goat IgG (A48257, Invitrogen) at concentrations 1:200 each. After incubation, the samples were rinsed with PBST and counterstained with propidium iodide (Sigma Aldrich) (5μg/ml in blocking buffer) in the dark for 10 min at room temperature, followed by rinsing with PBST and H_2O.

2. SUPPLEMENTARY TABLES

Supplementary Table S1. The primer sequences and annealing temperatures used in RT-qPCR for RNA-Seq data verification in rediscovery group.

Gene	Primer Sequence	Product Size	Annealing Temperature
LDHA	F: AGGCTACACATCCTGGGCTA<br>R: TTCAAACGGGCCTCTTCCTC	216 bp	59°C
RPL4	F: ATCCAAAGAGCCCTTCGAGC<br>R: GAGCTTGTGATTCCTGGCCT	156 bp	65°C
UBC	F: CCACTCTGCACTTGGTCCTG<br>R: TTGGGAATGCAACAACTTTAT	109 bp	59°C
TFRC	F: TGCTGCTTTCCCTTTCCTTG<br>R: CGTGCCACTTTGTTCAACTC	147 bp	65°C
MSMB	F: TGCACCCTTGTTTCTACACC<br>R: TGCAGTCCTCCTTCTTGAAG	73 bp	65°C
TLR2	F: TGGCCAGCAAATTACCTGTG<br>R: GGAACAGAGCACAGCACATG	172 bp	59°C

Supplementary Table S2. The detailed clinical data of examined KTCN and control individuals from the discovery group.
Clinical data concerning both eyes is presented. The eye with more advanced disease is indicated. Samples subjected to RNA-Seq and/or MALDI-TOF/TOF MS/MS assessment are annotated.

Patient ID	Diagnosis	Sex	Age	Examined eye	More advanced eye	K1 [D]	K2 [D]	Kmax [D]	Anterior Elevation [µm]	Posterior Elevation [µm]	TCT [µm]	TKC Grade	TET (in range 0.0-7.0mm) [µm]	Average thickness of *TR1* [µm]	Average thickness of *TR2* [µm]	Average thickness of *TR3* [µm]	Samples subjected to RNA-seq	Samples subjected to MALDI MS
9 KTCN	KTCN	M	18	OD	no	41	41.6	50.3	22	37	478	2	44	45	53	46	yes	yes
				OS	yes	40.8	41	52.1	24	46	473	2	43	44	52	47	yes	yes
10 KTCN	KTCN	M	14	OD	no	41.9	44.7	46.8	16	35	452	1-2 (1.5)	42	43	53	46	yes	yes
				OS	yes	49.5	54.8	66.2	39	52	391	3	34	35	57	47	yes	yes
14 KTCN	KTCN	M	17	OD	no	44.5	45.4	47	7	15	498	1-2 (1.5)	43	44	45	44	yes	yes
				OS	yes	44.3	46.8	53.6	12	31	462	2	39	41	51	44	yes	yes
22 KTCN	KTCN	M	25	OD	yes	43.1	47.2	52.5	18	40	480	2	46	46	53	48	yes	yes
				OS	no	42.4	46.5	52.4	19	42	484	2	46	46	53	48	yes	yes
32 KTCN	KTCN	M	42	OD	no	44.2	46.8	51.3	26	57	442	3	37	39	52	46	yes	yes
				OS	yes	47.7	51.1	53.3	32	65	407	3	39	40	52	46	yes	yes
41 KTCN	KTCN	M	19	OD	no	43	45.3	49.1	9	16	475	1-2 (1.5)	49	50	57	51	yes	yes
				OS	yes	45.4	50.3	56.9	22	54	463	2	44	44	57	50	yes	yes
64 KTCN	KTCN	M	23	OD	no	43.1	44.5	54.4	38	70	408	3	42	43	61	55	yes	yes
				OS	yes	50.9	52.8	61.3	48	83	364	3-4 (3.5)	37	39	60	55	yes	yes
67 KTCN	KTCN	M	21	OD	yes	44	48.1	60.4	50	90	420	3-4 (3.5)	35	43	55	50	yes	yes
				OS	no	45.2	49.2	61.7	44	73	430	3-4 (3.5)	38	42	52	50	yes	yes
75 KTCN	KTCN	M	22	OD	no	42.3	44.8	48.9	12	38	535	1-2 (1.5)	45	48	60	51	yes	yes
				OS	yes	43.9	50.8	59.7	33	75	501	3	41	44	58	51	yes	yes
80 KTCN	KTCN	M	26	OD	yes	41.7	44.9	52.2	30	66	471	3	42	45	68	53	yes	yes
				OS	no	40.7	41.8	51.4	32	71	485	3	46	49	69	54	yes	yes
82 KTCN	KTCN	M	24	OD	yes	56.2	66.9	84.1	58	164	319	3-4 (3.5)	39	48	60	50	yes	yes
				OS	no	43.2	45.4	50.4	18	39	534	2	36	50	60	50	yes	yes
87 KTCN	KTCN	M	14	OD	no	42.5	45.4	49	14	32	492	1-2 (1.5)	44	46	55	50	yes	yes
				OS	yes	42.5	45.8	49.6	14	33	495	1-2 (1.5)	44	46	54	49	yes	yes
97 KTCN	KTCN	M	19	OD	yes	42	45.6	51	17	36	500	2	40	44	55	51	yes	yes
				OS	no	41.3	42.9	46.1	13	29	508	1	53	54	60	55	yes	yes
102 KTCN	KTCN	M	18	OD	no	42.5	46.6	52.8	15	35	459	2	38	41	56	50	yes	yes
				OS	yes	50.5	56	69.3	52	93	408	3-4 (3.5)	38	41	57	50	yes	yes
135 KTCN	KTCN	M	17	OD	no	43.4	44.6	49	20	43	464	2	43	47	60	52	yes	yes

				OS	yes	51.4	54.6	66.9	59	106	424	3-4 (3.5)	46	42	62	55	yes	yes
140 KTCN	KTCN	M	27	OD	yes	44	48.8	57.3	31	55	449	3	42	44	55	50	yes	yes
				OS	no	43.4	47.4	54.9	25	45	453	2-3 (2.5)	44	45	56	50	yes	yes
143 KTCN	KTCN	M	26	OD	no	43.3	43.3	45.5	8	21	490	1-2 (1.5)	50	54	60	56	no	yes
				OS	yes	43.6	47.9	53.8	22	46	470	2	44	46	61	56	no	yes
151 KTCN	KTCN	M	23	OD	no	41.6	43.8	54	30	57	471	3	46	50	60	51	no	yes
				OS	yes	43.4	47.1	58.8	40	70	456	3-4 (3.5)	45	50	63	51	no	yes
154 KTCN	KTCN	M	27	OD	yes	42.1	44.1	48.3	14	37	452	1-2 (1.5)	50	51	60	50	no	yes
				OS	no	41.4	42.3	43.4	6	12	469	FF	50	52	56	51	no	yes
161 KTCN	KTCN	M	22	OD	no	44.8	45.4	50	14	29	503	1-2 (1.5)	45	49	61	51	no	yes
				OS	yes	53.6	53.9	70.3	53	94	449	3-4 (3.5)	49	51	65	53	no	yes
166 KTCN	KTCN	M	23	OD	no	44	47	51.4	15	43	470	1-2 (1.5)	47	49	57	52	no	yes
				OS	yes	53.6	59.8	71.4	43	81	411	3	40	45	59	52	no	yes
1 M	CONTROL	F	33	OD	yes	45.4	46.5	47	2	-2	501	-	45	48	47	45	yes	yes
				OS	no	45.4	46.3	46.7	1	-1	507	-	44	52	48	47	yes	yes
4 M	CONTROL	F	30	OD	yes	44	44.8	45.2	0	-1	515	-	41	48	45	45	yes	yes
				OS	no	44.4	45.3	45.7	1	4	516	-	41	46	44	44	yes	yes
5 M	CONTROL	M	24	OD	no	42.9	43.9	44.3	2	2	539	-	46	51	49	48	yes	yes
				OS	yes	43.3	44.2	44.4	2	2	531	-	46	52	50	49	yes	yes
6 M	CONTROL	M	30	OD	yes	41.3	42.8	43.1	1	1	489	-	45	49	46	45	yes	yes
				OS	no	41.5	43.1	43.4	0	1	476	-	44	49	46	45	yes	yes
10 M	CONTROL	F	42	OD	no	45.2	46.3	46.8	1	5	527	-	46	55	51	48	yes	yes
				OS	yes	45.5	46.3	46.7	1	5	533	-	47	54	50	48	yes	yes
11 M	CONTROL	F	23	OD	no	42.7	44.6	45	2	10	592	-	48	54	54	50	yes	yes
				OS	yes	43	44.5	44.8	3	7	594	-	49	55	53	50	yes	yes

Abbreviations in table: D – diopters, F – female, K1 – flat keratometry, K2 – steep keratometry, Kmax – maximal corneal curvature, KTCN- keratoconus, M – male, MALDI-MS – tandem matrix-assisted laser desorption/ionization-time of flight/time of flight mass spectrometry, OD – right eye, OS – left eye, TCT – thinnest corneal thickness, TET – thinnest epithelial thickness, TKC – Topographic Keratoconus Classification, *TR1 – central topographic region*, *TR2 – middle topographic region*, *TR3 – peripheral topographic region* .

Table S3. The results of the quality and quantity control of RNA samples.

The results of quality (Nanodrop ND-1000) and quantity of the purified RNA (RNA 6000 Nano Kit, Agilent Technologies) and the number of RNAseq read before and after quality control (including rRNA depletion) for each analyzed sample. After rRNA depletion, we obtained an average of 89.9 million reads per sample, ranging from 48.6 to 171.3 million reads.

No.	Sample ID	Patient ID	*TR* of CE	RNA concentration [ng/ul]	260/280 ratio	DNase I treatment	RIN values	Total RNA input [ng]	Total reads	No rRNA trimmed_reads
1	10 KTCN/OS/1	10 KTCN	*TR1*	91.60	2.09	yes	7.9	630.0	145085043	139454613
2	10 KTCN/OS/2	10 KTCN	*TR2*	244.37	2.10	yes	8	812.0	127494040	102227812
3	10 KTCN/OS/3	10 KTCN	*TR3*	426.45	2.06	yes	7.4	788.9	129114226	127388304
4	10 KTCN/OD/1	10 KTCN	*TR1*	121.15	2.03	yes	7.6	800.3	81662853	76974569
5	10 KTCN/OD/2	10 KTCN	*TR2*	123.03	2.04	yes	7.7	798.0	96774421	84643044
6	10 KTCN/OD/3	10 KTCN	*TR3*	237.83	2.03	yes	7.5	797.5	79269100	72954976
7	14 KTCN/OS/1	14 KTCN	*TR1*	85.77	2.07	yes	6.3	740.0	134946999	129298105
8	14 KTCN/OS/2	14 KTCN	*TR2*	189.09	2.11	yes	6.7	803.6	123455689	120385846
9	14 KTCN/OS/3	14 KTCN	*TR3*	431.28	2.09	yes	6.2	797.5	128232392	121521367
10	14 KTCN/OD/1	14 KTCN	*TR1*	54.97	1.99	yes	8.7	470.0	96403395	92101282
11	14 KTCN/OD/2	14 KTCN	*TR2*	205.90	2.02	yes	8.5	808.0	71925175	67581989
12	14 KTCN/OD/3	14 KTCN	*TR3*	256.60	2.02	yes	8.6	808.6	103013893	84348774
13	22 KTCN/OS/1	22 KTCN	*TR1*	13.75	1.86	yes	5.7	55.0	74839657	63001315
14	22 KTCN/OS/2	22 KTCN	*TR2*	116.55	2.02	yes	7.8	806.4	74407534	72023265
15	22 KTCN/OS/3	22 KTCN	*TR3*	467.20	2.01	yes	7.8	814.4	88815381	84624470
16	22 KTCN/OD/1	22 KTCN	*TR1*	69.10	2.14	yes	9.4	430.0	119076146	108579045
17	22 KTCN/OD/2	22 KTCN	*TR2*	137.87	2.10	yes	8.7	796.8	135849671	129125558
18	22 KTCN/OD/3	22 KTCN	*TR3*	372.23	2.09	yes	8.4	801.0	129924062	128153726
19	32 KTCN/OS/1	32 KTCN	*TR1*	79.57	2.09	yes	8.4	795.4	129466985	125407374
20	32 KTCN/OS/2	32 KTCN	*TR2*	560.31	2.05	yes	8.1	813.4	163469075	157827889
21	32 KTCN/OS/3	32 KTCN	*TR3*	217.29	2.08	yes	7.3	800.0	184640247	151208331
22	32 KTCN/OD/1	32 KTCN	*TR1*	86.29	1.95	yes	8.7	792.0	67033427	65266883
23	32 KTCN/OD/2	32 KTCN	*TR2*	75.76	1.99	yes	8.2	801.0	71214428	68910793
24	32 KTCN/OD/3	32 KTCN	*TR3*	606.61	2.04	yes	7.6	815.0	63264605	60413485
25	41 KTCN/OS/1	41 KTCN	*TR1*	77.72	1.97	yes	4.9	215.6	66412838	60663293
26	41 KTCN/OS/2	41 KTCN	*TR2*	178.54	2.01	yes	4.9	792.0	126476387	121754127
27	41 KTCN/OS/3	41 KTCN	*TR3*	248.46	2.02	yes	3.9	786.6	81408081	78700185
28	41 KTCN/OD/1	41 KTCN	*TR1*	39.26	1.97	yes	8.5	156.0	98820860	93980945
29	41 KTCN/OD/2	41 KTCN	*TR2*	226.99	2.01	yes	8.6	800.0	70601909	62903985
30	41 KTCN/OD/3	41 KTCN	*TR3*	361.56	2.02	yes	8.4	818.4	110530963	72887782
31	64 KTCN/OS/1	64 KTCN	*TR1*	59.38	1.97	yes	8.5	530.0	139022888	136368954
32	64 KTCN/OS/2	64 KTCN	*TR2*	106.10	1.97	yes	8.7	804.0	158385386	155675331

33	64 KTCN/OS/3	64 KTCN	*TR3*	220.53	2.05	yes	8.6	812.7	64309504	61992139
34	64 KTCN/OD/1	64 KTCN	*TR1*	64.32	2.02	yes	8.9	760.0	64109626	62664010
35	64 KTCN/OD/2	64 KTCN	*TR2*	57.33	2.00	yes	8.9	470.0	87121668	84909597
36	64 KTCN/OD/3	64 KTCN	*TR3*	170.66	2.01	yes	8.5	792.0	162360698	148830327
37	67 KTCN/OS/1	67 KTCN	*TR1*	41.12	1.99	yes	9.2	450.0	100343634	97780076
38	67 KTCN/OS/2	67 KTCN	*TR2*	93.41	2.00	yes	9.2	670.0	71227288	68716981
39	67 KTCN/OS/3	67 KTCN	*TR3*	317.11	2.04	yes	8.9	815.1	64282406	62993608
40	67 KTCN/OD/1	67 KTCN	*TR1*	32.07	1.90	yes	9.5	370.0	67313180	65232976
41	67 KTCN/OD/2	67 KTCN	*TR2*	62.00	2.03	yes	9.1	690.0	154180946	125134295
42	67 KTCN/OD/3	67 KTCN	*TR3*	182.12	2.05	yes	8.7	806.4	55789707	61944160
43	75 KTCN/OS/1	75 KTCN	*TR1*	68.20	1.99	yes	9.1	730.0	74075202	68224680
44	75 KTCN/OS/2	75 KTCN	*TR2*	71.87	2.02	yes	9.1	690.0	83963145	79249196
45	75 KTCN/OS/3	75 KTCN	*TR3*	125.75	2.00	yes	8.9	806.0	85125789	80744063
46	75 KTCN/OD/1	75 KTCN	*TR1*	31.94	1.95	yes	8.8	350.0	110658473	74578729
47	75 KTCN/OD/2	75 KTCN	*TR2*	39.22	1.99	yes	9.1	360.0	72613067	68893654
48	75 KTCN/OD/3	75 KTCN	*TR3*	167.27	2.08	yes	9.1	806.4	162772418	157969791
49	80 KTCN/OS/1	80 KTCN	*TR1*	106.20	2.04	yes	9.1	802.4	101405747	64425114
50	80 KTCN/OS/2	80 KTCN	*TR2*	142.08	2.05	yes	8.9	815.1	83130921	79082325
51	80 KTCN/OS/3	80 KTCN	*TR3*	222.44	2.05	yes	9	800.8	74232016	70365324
52	80 KTCN/OD/1	80 KTCN	*TR1*	102.04	2.03	yes	9.1	804.0	88668215	64772911
53	80 KTCN/OD/2	80 KTCN	*TR2*	132.81	2.04	yes	9.1	760.5	89022829	48573601
54	80 KTCN/OD/3	80 KTCN	*TR3*	232.46	2.05	yes	8.9	811.2	87859349	83740139
55	82 KTCN/OS/1	82 KTCN	*TR1*	47.48	1.99	yes	9.1	740.0	78895346	63237353
56	82 KTCN/OS/2	82 KTCN	*TR2*	43.09	1.93	yes	8.9	799.8	66858161	64732679
57	82 KTCN/OS/3	82 KTCN	*TR3*	113.21	2.02	yes	9.1	794.5	135078222	51862248
58	82 KTCN/OD/1	82 KTCN	*TR1*	47.57	1.99	yes	8.8	790.0	67315303	63491484
59	82 KTCN/OD/2	82 KTCN	*TR2*	81.92	2.00	yes	8.9	798.6	67967216	61037953
60	82 KTCN/OD/3	82 KTCN	*TR3*	43.96	1.98	yes	9	660.0	168597319	161125195
61	87 KTCN/OS/1	87 KTCN	*TR1*	38.65	2.00	yes	8.4	610.0	131067971	70559467
62	87 KTCN/OS/2	87 KTCN	*TR2*	41.03	1.92	yes	8.6	620.0	71261532	68619927
63	87 KTCN/OS/3	87 KTCN	*TR3*	177.53	2.05	yes	8.9	813.2	158221703	149719395
64	87 KTCN/OD/1	87 KTCN	*TR1*	35.37	1.89	yes	8.1	400.0	83846455	80047998
65	87 KTCN/OD/2	87 KTCN	*TR2*	46.61	1.91	yes	8.2	430.0	153641883	68845875
66	87 KTCN/OD/3	87 KTCN	*TR3*	224.77	2.04	yes	8.8	807.0	78517770	73294390
67	9 KTCN/OS/1	9 KTCN	*TR1*	85.88	2.10	yes	6.5	799.2	148030333	143077513
68	9 KTCN/OS/2	9 KTCN	*TR2*	107.07	2.12	yes	6.8	803.6	132649024	111679824
69	9 KTCN/OS/3	9 KTCN	*TR3*	297.35	2.10	yes	6.3	811.2	149679722	142956889
70	9 KTCN/OD/1	9 KTCN	*TR1*	158.07	2.02	yes	9	804.1	73258306	70353461
71	9 KTCN/OD/2	9 KTCN	*TR2*	113.59	1.98	yes	8.4	799.8	91184402	87612295
72	9 KTCN/OD/3	9 KTCN	*TR3*	441.97	2.02	yes	8.2	804.7	91985752	86363316

73	97 KTCN/OS/1	97 KTCN	*TR1*	29.95	2.02	yes	8.8	450.0	90072708	87268439
74	97 KTCN/OS/2	97 KTCN	*TR2*	70.30	1.97	yes	9	797.9	97523258	88742636
75	97 KTCN/OS/3	97 KTCN	*TR3*	182.17	2.05	yes	8.7	817.8	93242233	85392950
76	97 KTCN/OD/1	97 KTCN	*TR1*	28.96	1.91	yes	8.9	290.0	72175130	68208856
77	97 KTCN/OD/2	97 KTCN	*TR2*	47.04	2.00	yes	8.6	590.0	84043900	80276528
78	97 KTCN/OD/3	97 KTCN	*TR3*	300.15	2.05	yes	7.7	810.0	98132225	94525563
79	102 KTCN/OS/1	102 KTCN	*TR1*	37.87	1.98	yes	8.5	450.0	88458270	85663097
80	102 KTCN/OS/2	102 KTCN	*TR2*	132.65	2.03	yes	8.5	814.5	77457386	74215584
81	102 KTCN/OS/3	102 KTCN	*TR3*	307.27	2.06	yes	9	792.0	181419102	171286414
82	102 KTCN/OD/1	102 KTCN	*TR1*	34.31	1.98	yes	7.4	340.0	75730398	72481019
83	102 KTCN/OD/2	102 KTCN	*TR2*	47.16	2.01	yes	7.8	650.0	70938691	63873996
84	102 KTCN/OD/3	102 KTCN	*TR3*	280.38	2.06	yes	8.2	795.6	84194838	67884675
85	135 KTCN/OS/1	135 KTCN	*TR1*	28.76	1.92	yes	9	400.0	76788761	73100156
86	135 KTCN/OS/2	135 KTCN	*TR2*	151.23	2.04	yes	8.6	796.4	99238389	95857895
87	135 KTCN/OS/3	135 KTCN	*TR3*	99.18	2.00	yes	8.7	800.8	113451453	77414242
88	135 KTCN/OD/1	135 KTCN	*TR1*	30.42	2.05	yes	8.6	250.0	71759282	68773643
89	135 KTCN/OD/2	135 KTCN	*TR2*	139.44	2.03	yes	8.6	796.5	77355693	64805837
90	135 KTCN/OD/3	135 KTCN	*TR3*	231.01	2.03	yes	8.8	802.8	77125035	64323540
91	140 KTCN/OS/1	140 KTCN	*TR1*	52.90	1.97	yes	8.8	630.0	67412906	61454720
92	140 KTCN/OS/2	140 KTCN	*TR2*	121.34	2.03	yes	8.2	795.0	81317367	74540212
93	140 KTCN/OS/3	140 KTCN	*TR3*	253.85	2.03	yes	8.2	800.8	83736394	79249409
94	140 KTCN/OD/1	140 KTCN	*TR1*	43.42	1.95	yes	8	630.0	72278183	67280623
95	140 KTCN/OD/2	140 KTCN	*TR2*	104.77	2.04	yes	8.3	805.0	76735915	73152043
96	140 KTCN/OD/3	140 KTCN	*TR3*	378.37	2.03	yes	7.7	816.0	72710293	69060332
97	1 M/OS/1	1 M	*TR1*	115.33	2.00	yes	8.2	810.9	101882519	80309283
98	1 M/OS/2	1 M	*TR2*	200.82	2.03	yes	7.9	811.2	71644110	67959339
99	1 M/OS/3	1 M	*TR3*	499.89	2.08	yes	7.9	798.0	74897811	71539319
100	1 M/OD/1	1 M	*TR1*	131.82	2.09	yes	8	804.0	172934970	168824960
101	1 M/OD/2	1 M	*TR2*	302.39	2.09	yes	8.2	810.0	83307794	81000364
102	1 M/OD/3	1 M	*TR3*	391.56	2.09	yes	8.2	825.7	177917811	167701580
103	10 M/OS/1	10 M	*TR1*	39.38	1.87	yes	5.5	496.0	118285021	113555832
104	10 M/OS/2	10 M	*TR2*	86.94	1.94	yes	4	802.3	78537489	76323055
105	10 M/OS/3	10 M	*TR3*	222.81	2.02	yes	4.1	813.0	67140794	64008692
106	10 M/OD/1	10 M	*TR1*	18.42	1.94	yes	5.4	225.0	79401052	59983381
107	10 M/OD/2	10 M	*TR2*	99.91	1.97	yes	4.3	799.8	82830681	79022897
108	10 M/OD/3	10 M	*TR3*	277.55	2.03	yes	4.9	819.0	130834175	125362985
109	11 M/OS/1	11 M	*TR1*	49.56	1.92	yes	5.4	540.0	80223595	77225574
110	11 M/OS/2	11 M	*TR2*	77.28	1.96	yes	5	780.0	122518566	116706801
111	11 M/OS/3	11 M	*TR3*	138.90	2.00	yes	5	800.3	107381958	104078028
112	11 M/OD/1	11 M	*TR1*	34.08	1.82	yes	4.6	320.0	70908921	67817965

113	11 M/OD/2	11 M	*TR2*	59.98	1.99	yes	3.2	720.0	73189089	69983192
114	11 M/OD/3	11 M	*TR3*	152.96	2.05	yes	4.1	806.4	75887656	65000013
115	4 M/OS/1	4 M	*TR1*	95.37	2.06	yes	8.7	805.0	78531685	72616233
116	4 M/OS/2	4 M	*TR2*	196.61	2.05	yes	9.2	812.0	88312300	70509627
117	4 M/OS/3	4 M	*TR3*	460.48	1.99	yes	8.2	802.1	67410671	64690536
118	4 M/OD/1	4 M	*TR1*	96.96	2.09	yes	6.6	640.0	145402612	135147738
119	4 M/OD/2	4 M	*TR2*	202.50	2.09	yes	6.9	809.2	141267531	135765833
120	4 M/OD/3	4 M	*TR3*	552.97	2.08	yes	8.5	835.9	122707897	116871083
121	5 M/OS/1	5 M	*TR1*	62.63	2.09	yes	7.1	816.0	113219818	108983944
122	5 M/OS/2	5 M	*TR2*	152.31	2.08	yes	7.1	800.8	111430762	107650394
123	5 M/OS/3	5 M	*TR3*	392.12	2.08	yes	7.7	811.2	105965206	103294222
124	5 M/OD/1	5 M	*TR1*	45.80	1.91	yes	8.5	580.0	66603591	61319599
125	5 M/OD/2	5 M	*TR2*	163.55	2.05	yes	8.3	815.1	76271406	63426651
126	5 M/OD/3	5 M	*TR3*	496.80	2.10	yes	8.4	771.1	66452129	61856751
127	6 M/OS/1	6 M	*TR1*	68.87	2.06	yes	6.9	670.0	90711882	62282334
128	6 M/OS/2	6 M	*TR2*	143.20	2.09	yes	7.2	793.8	65243160	62725328
129	6 M/OS/3	6 M	*TR3*	125.05	2.10	yes	6.5	806.0	91362069	72891558
130	6 M/OD/1	6 M	*TR1*	117.61	2.01	yes	8.8	792.0	166892756	152226542
131	6 M/OD/2	6 M	*TR2*	221.17	2.03	yes	8.4	808.8	121383060	119608259
132	6 M/OD/3	6 M	*TR3*	414.61	2.00	yes	8.4	811.2	221452408	117868501

Abbreviations: KTCN – keratoconus, M – myopia, OD – right eye, OS – left eyes, *TR – topographic region*, *TR1 – central topographic region*, *TR2 – middle topographic region*, *TR3 – peripheral topographic region,* RIN – RNA Integrity Number

Supplementary Table S4. Differentially expressed genes (DEGs) in paired model of KTCN.

Differential expression analysis was performed using limma package(Law et al., 2018; Ritchie et al., 2015) in two settings: I) patients with KTCN whose inter-eye asymmetry expressed in the TKC grades was ≥1 (*n*=6 pairs), and II) patients with KTCN whose inter-eye asymmetry expressed in the TKC grades was >1 (*n*=5 pairs). For each *topographic region* (*central*/*middle*/*peripheral TR*) and each setting, analysis was performed separately. Analysis input (particular *topographic region* and setting I or II), Ensembl gene identifier ('Gene ID'), gene name, gene biotype, log2FC, p-values, and adjusted p-values are indicated. P-values were adjusted for multiple comparisons using the Benjamini-Hochberg correction method.

Analysis input	Gene ID	Gene name	Gene Biotype	Log2FC	AveExpr	t	P.Value	adj.P.Value	B
central TR, setting I	ENSG00000179023	*KLHDC7A*	protein_coding	1.8823	-0.4726	5.5840	1.35E-07	0.0035	6.3412
central TR, setting I	ENSG00000241679	*AC018450.1*	lncRNA	1.7176	-2.0850	5.1064	1.16E-06	0.0150	3.9554
central TR, setting I	ENSG00000175040	*CHST2*	protein_coding	1.5704	3.7644	4.7819	4.7E-06	0.0294	3.9310
central TR, setting I	ENSG00000141404	*GNAL*	protein_coding	-0.6274	6.1783	-4.8240	3.93E-06	0.0294	4.0807
central TR, setting I	ENSG00000251637	*LINC02754*	lncRNA	0.8848	3.3765	4.5661	1.15E-05	0.0372	3.1174
central TR, setting I	ENSG00000132429	*POPDC3*	protein_coding	2.3370	-2.6497	4.6033	9.89E-06	0.0372	2.0892
central TR, setting I	ENSG00000114251	*WNT5A*	protein_coding	0.6143	4.4169	4.5848	1.07E-05	0.0372	3.1751
central TR, setting I	ENSG00000283633	*AP000547.3*	lncRNA	-0.9888	1.5595	-4.5138	1.43E-05	0.0410	2.7917
central TR, setting II	ENSG00000182771	*GRID1*	protein_coding	1.7750	-0.1538	5.6368	1.05E-07	0.0027	7.1097
central TR, setting II	ENSG00000141404	*GNAL*	protein_coding	-0.7168	6.1783	-5.3801	3.42E-07	0.0033	6.3558
central TR, setting II	ENSG00000114251	*WNT5A*	protein_coding	0.7156	4.4169	5.3535	3.86E-07	0.0033	6.2543
central TR, setting II	ENSG00000071205	*ARHGAP10*	protein_coding	0.5229	4.2171	5.2647	5.76E-07	0.0037	5.8830
central TR, setting II	ENSG00000105523	*FAM83E*	protein_coding	0.9278	2.2464	5.0666	1.38E-06	0.0052	5.0275
central TR, setting II	ENSG00000139289	*PHLDA1*	protein_coding	1.3883	1.1846	5.1273	1.06E-06	0.0052	5.1574
central TR, setting II	ENSG00000105851	*PIK3CG*	protein_coding	1.4599	0.6965	5.0620	1.41E-06	0.0052	4.9176
central TR, setting II	ENSG00000111405	*ENDOU*	protein_coding	1.0504	-0.2913	4.9321	2.48E-06	0.0064	4.2532
central TR, setting II	ENSG00000179023	*KLHDC7A*	protein_coding	1.7899	-0.4726	4.9659	2.14E-06	0.0064	4.2886
central TR, setting II	ENSG00000178695	*KCTD12*	protein_coding	1.4392	-0.4360	4.8766	3.14E-06	0.0074	4.0060
central TR, setting II	ENSG00000276180	*H4C9*	protein_coding	1.5024	-0.6752	4.5369	1.3E-05	0.0258	2.7738
central TR, setting II	ENSG00000145384	*FABP2*	protein_coding	1.1698	0.5466	4.4948	1.54E-05	0.0266	2.7440
central TR, setting II	ENSG00000186732	*MPPED1*	protein_coding	-0.8337	2.6228	-4.4950	1.54E-05	0.0266	2.8419
central TR, setting II	ENSG00000155380	*SLC16A1*	protein_coding	0.9422	1.5010	4.4759	1.66E-05	0.0269	2.7544
central TR, setting II	ENSG00000275678	*AL133320.2*	lncRNA	-1.3465	-1.9845	-4.4110	2.16E-05	0.0294	1.1754
central TR, setting II	ENSG00000170017	*ALCAM*	protein_coding	1.0308	1.5281	4.4256	2.04E-05	0.0294	2.5905
central TR, setting II	ENSG00000119888	*EPCAM*	protein_coding	0.5411	2.9729	4.4126	2.15E-05	0.0294	2.5488
central TR, setting II	ENSG00000224914	*LINC00863*	lncRNA	-0.5343	4.5361	-4.3808	2.44E-05	0.0301	2.4010
central TR, setting II	ENSG00000165194	*PCDH19*	protein_coding	1.5566	0.2907	4.3803	2.44E-05	0.0301	2.3554
central TR, setting II	ENSG00000273356	*LINC02019*	lncRNA	0.7978	-0.6657	4.3328	2.95E-05	0.0318	1.9779
central TR, setting II	ENSG00000072274	*TFRC*	protein_coding	0.5798	9.3413	4.3327	2.95E-05	0.0318	2.0975
central TR, setting II	ENSG00000073008	*PVR*	protein_coding	0.7043	1.9718	4.3167	3.14E-05	0.0325	2.2034
central TR, setting II	ENSG00000186212	*SOWAHB*	protein_coding	0.5977	2.5777	4.2944	3.43E-05	0.0341	2.1251
central TR, setting II	ENSG00000250091	*DNAH10OS*	lncRNA	-0.6567	2.0535	-4.2847	3.57E-05	0.0341	2.0695

central TR, setting II	ENSG00000112414	*ADGRG6*	protein_coding	1.0219	5.0927	4.2388	4.27E-05	0.0368	1.8343
central TR, setting II	ENSG00000187094	*CCK*	protein_coding	1.2559	-0.6313	4.2402	4.25E-05	0.0368	1.7956
central TR, setting II	ENSG00000066248	*NGEF*	protein_coding	1.0702	2.0010	4.2459	4.15E-05	0.0368	1.9549
central TR, setting II	ENSG00000263639	*MSMB*	protein_coding	1.5936	3.2974	4.2174	4.64E-05	0.0375	1.8306
central TR, setting II	ENSG00000188730	*VWC2*	protein_coding	1.3635	1.0708	4.2185	4.62E-05	0.0375	1.8548
central TR, setting II	ENSG00000170989	*S1PR1*	protein_coding	0.5862	4.3663	4.1807	5.35E-05	0.0395	1.6562
central TR, setting II	ENSG00000167216	*KATNAL2*	protein_coding	-0.6232	2.0545	-4.1710	5.55E-05	0.0399	1.6783
central TR, setting II	ENSG00000125266	*EFNB2*	protein_coding	0.7208	2.2185	4.1478	6.08E-05	0.0424	1.6133
central TR, setting II	ENSG00000072110	*ACTN1*	protein_coding	0.6381	3.6811	4.1306	6.49E-05	0.0441	1.5099
central TR, setting II	ENSG00000262714	*AC007342.5*	lncRNA	1.0750	0.0194	4.1163	6.85E-05	0.0454	1.4130
central TR, setting II	ENSG00000176595	*KBTBD11*	protein_coding	0.7690	2.6044	4.0815	7.83E-05	0.0494	1.3821
central TR, setting II	ENSG00000183379	*SYNDIG1L*	protein_coding	0.9225	-1.5780	4.0850	7.72E-05	0.0494	1.0865
central TR, setting II	ENSG00000178919	*FOXE1*	protein_coding	1.8865	-2.3982	4.0750	8.02E-05	0.0494	0.7678
middle TR, setting I	ENSG00000141404	*GNAL*	protein_coding	-0.7215	6.1783	-5.5617	1.49E-07	0.0039	6.9663
middle TR, setting I	ENSG00000179023	*KLHDC7A*	protein_coding	1.8706	-0.4726	5.3985	3.15E-07	0.0041	4.0250
middle TR, setting II	ENSG00000141404	*GNAL*	protein_coding	-0.7759	6.1783	-5.8348	4.17E-08	0.0011	7.9909
middle TR, setting II	ENSG00000105523	*FAM83E*	protein_coding	1.0172	2.2464	5.6601	9.46E-08	0.0012	6.4213
middle TR, setting II	ENSG00000263639	*MSMB*	protein_coding	1.9729	3.2974	5.5566	1.53E-07	0.0013	6.6103
middle TR, setting II	ENSG00000102048	*ASB9*	protein_coding	1.0734	-1.1630	5.2990	4.94E-07	0.0032	2.9435
peripheral TR, setting I	ENSG00000260273	*AL359711.2*	lncRNA	0.9905	-0.2511	5.3927	3.24E-07	0.0084	6.0112
peripheral TR, setting I	ENSG00000179023	*KLHDC7A*	protein_coding	1.7031	-0.4726	5.0646	1.39E-06	0.0180	4.7171
peripheral TR, setting I	ENSG00000274333	*CU633967.1*	lncRNA	-1.6939	2.1495	-4.8095	4.18E-06	0.0216	4.0198
peripheral TR, setting I	ENSG00000277991	*FP236241.2*	lncRNA	-2.3280	0.7759	-4.8258	3.9E-06	0.0216	3.8725
peripheral TR, setting I	ENSG00000141404	*GNAL*	protein_coding	-0.6263	6.1783	-4.9150	2.67E-06	0.0216	4.3828
peripheral TR, setting II	ENSG00000105523	*FAM83E*	protein_coding	1.0036	2.2464	5.8603	3.69E-08	0.0010	8.4215
peripheral TR, setting II	ENSG00000263639	*MSMB*	protein_coding	1.9496	3.2974	5.6545	9.71E-08	0.0013	7.5501
peripheral TR, setting II	ENSG00000137462	*TLR2*	protein_coding	0.6073	3.2904	5.2140	7.22E-07	0.0062	5.6698
peripheral TR, setting II	ENSG00000141404	*GNAL*	protein_coding	-0.6579	6.1783	-5.0546	1.46E-06	0.0094	4.9520
peripheral TR, setting II	ENSG00000260273	*AL359711.2*	lncRNA	0.9651	-0.2511	4.8683	3.26E-06	0.0168	4.0636
peripheral TR, setting II	ENSG00000179023	*KLHDC7A*	protein_coding	1.6927	-0.4726	4.7297	5.85E-06	0.0252	3.5477
peripheral TR, setting II	ENSG00000277991	*FP236241.2*	lncRNA	-2.4865	0.7759	-4.6241	9.08E-06	0.0293	3.1030
peripheral TR, setting II	ENSG00000188710	*QRFP*	protein_coding	0.9985	2.0216	4.4502	1.85E-05	0.0477	2.6862

Abbreviations: log2FC – log2 fold change, setting I – patients with KTCN whose inter-eye asymmetry expressed in the TKC grades was ≥1, setting II – patients with KTCN whose inter-eye asymmetry expressed in the TKC grades was >1, *TR – topographic region*

Supplementary Table S5. Discriminative proteins in paired model of KTCN.

Identified proteins classified as discriminative in paired analysis model for particular *topographic regions* in three settings: I) all patients with KTCN (*n*=36 pairs), II) patients with KTCN whose inter-eye asymmetry expressed in the Topographic Keratoconus Classification (TKC) grades was ≥1 (*n*=8 pairs), and II) patients with KTCN whose inter-eye asymmetry expressed in the TKC grades was >1 (*n*=7 pairs). For each *topographic region* (*central*/*middle*/*peripheral TR*) and each setting, analysis was performed separately. The analysis input (particular *topographic region* and setting I/II/III), m/z values, fragment sequence, gene name, and p-values of the Wilcoxon signed-rank test are presented.

			Analysis input								
m/z value	Fragment sequence	Gene	*central TR,* setting I	*central TR,* setting II	*central TR,* setting III	*middle TR,* setting I	*middle TR,* setting II	*middle TR,* setting III	*peripheral TR,* setting I	*peripheral TR,* setting II	*peripheral TR,* setting III
918.5186993	R.RTTASLLR.K	*MTERFD2*	0.096	0.359	0.461	0.588	0.734	0.742	0.003	0.301	0.383
1009.508715	R.SYDEAILR.L	*BTN2A2*	0.926	0.726	0.8	0.56	0.554	0.554	0.288	0.008	0.016
1099.613748	R.IQQLEALQR.L	*ALDH3A1*	0.112	0.529	0.673	0.459	0.933	1	0.001	0.08	0.052
1142.535865	K.EELDQQNKR.F	*GOLGA4*	0.518	0.933	1	1	0.402	0.59	0.542	0.039	0.008
1153.579693	K.IREWYETR.G	*KRT12*	0.011	0.129	0.078	0.49	0.91	0.844	0.007	0.02	0.008
1154.577639	K.IREWYETR.G	*KRT12*	0.004	0.08	0.076	0.317	0.673	0.402	0.061	0.08	0.078
1165.606688	R.LFDQAFGLPR.L	*HSPB1*	0.001	0.039	0.039	0.658	0.25	0.25	0.003	0.004	0.008
1166.60228	R.LFDQAFGLPR.L	*HSPB1*	0.022	0.203	0.148	0.178	0.834	0.933	0.004	0.055	0.023
1197.663686	R.LGCGKGVPDPR.S	*RELN*	0.082	0.57	0.313	0.812	0.012	0.008	0.02	0.25	0.148
1263.689918	K.LALDVEIATYR.K	*KRT3*	0.026	0.301	0.313	0.495	0.734	0.844	0.003	0.129	0.109
1264.692268	K.LALDVEIATYR.K	*KRT3*	0.026	0.203	0.25	0.128	0.82	0.742	0.003	0.359	0.383
1265.692258	K.LALDVEIATYR.K	*KRT3*	0.002	0.076	0.142	0.173	0.726	0.933	0.024	0.164	0.078
1322.655187	K.NRSQGNIISYK.R	*TMEM217*	0.065	0.734	0.844	0.243	0.652	0.844	0.001	0.021	0.022
1323.650852	K.NRSQGNIISYK.R	*TMEM217*	0.026	0.203	0.25	0.205	0.353	0.402	0.009	0.208	0.059
1340.702261	R.LKSDVALEVPPK.R	*HELLS*	0.082	0.426	0.641	0.076	0.203	0.148	0.005	0.098	0.148
1392.749705	R.LVWILGRGGSHR.R	*FFAR3*	0.011	0.652	0.844	0.838	0.129	0.055	0.0009	0.203	0.109
1393.740482	K.YLLKEDMAGIPK.A	*FAR2*	0.004	0.82	0.844	0.288	0.652	0.461	0.05	0.82	1
1517.730027	K.IWHHTFYNELR.V	*ACTB*	0.01	0.039	0.078	0.338	0.554	0.675	0.051	0.496	0.25
1779.921335	K.AVITVQRPGRIPTSSR.C	*LAMB1*	0.008	0.129	0.109	0.896	0.294	0.205	0.026	0.164	0.109
1791.89342	x		0.006	0.074	0.109	0.147	1	0.742	0.412	0.734	0.547
1792.895859	x		0.006	0.25	0.461	0.658	0.203	0.109	0.07	0.301	0.148
1796.852034	x		0.422	0.496	0.195	0.009	0.673	0.402	0.931	1	0.673
1885.949555	K.STVSSLLQKEICPLIR.I	*BPIFA2*	0.009	0.652	0.844	0.257	0.82	0.945	0.046	0.301	0.109

1907.981268	K.QDLIKMTAILTTDVSDK.A	*ANK2*	0.288	0.734	0.313	1	0.359	0.195	0.168	0.008	0.008
1918.904873	x		0.003	0.129	0.023	0.296	0.624	0.8	0.478	0.272	0.272
1988.006537	x		0.892	0.82	0.945	0.006	0.301	0.25	0.243	0.652	1
1989.006468	M.VNPTVFFDIAVDGEPLGR.V	*PPIA*	0.006	0.074	0.039	0.024	0.02	0.039	0.003	0.027	0.055
2034.043454	R.LLQQLAMTGSEEGDPRTK.S	*GAPVD1*	0.035	0.82	0.945	0.36	0.944	0.933	0.005	0.359	0.195
2041.081135	M.AGPRVEVDGSIMEGGGQILR.V	*RTCD1*	0.002	0.426	0.742	0.632	0.944	0.8	0.033	0.183	0.022
2042.079746	M.AGPRVEVDGSIMEGGGQILR.V	*RTCD1*	0.002	0.129	0.25	0.387	0.933	0.834	0.103	0.529	0.272
2056.006268	K.VVLGINNLDHPSVFMQTR.F	*CORIN*	0.008	0.359	0.461	0.089	1	0.547	0.013	0.359	0.148
2058.008235	K.VVLGINNLDHPSVFMQTR.F	*CORIN*	0.006	0.426	0.383	0.157	0.652	0.25	0.008	0.25	0.078
2059.011509	x		0.06	0.57	0.461	0.338	0.496	0.25	0.004	0.129	0.055
2194.073055	R.CPFDRQVTDLGDSGVWGLK.K	*TRIM23*	0.0009	0.012	0.023	0.065	0.933	0.675	0.108	1	0.675
2297.138205	x		0.865	0.82	0.383	0.006	0.129	0.078	0.412	0.57	0.547
2331.054414	x		0.203	0.004	0.008	0.287	0.441	0.8	0.355	0.734	0.844
2371.229325	M.ACPLDQAIGLLVAIFHKYSGR.E	*S100A6*	0.103	0.496	0.461	0.006	0.164	0.313	0.119	0.652	0.547
2372.230212	M.ACPLDQAIGLLVAIFHKYSGR.E	*S100A6*	0.051	0.529	0.447	0.008	0.203	0.383	0.137	0.652	0.461
2373.240027	M.ACPLDQAIGLLVAIFHKYSGR.E	*S100A6*	0.127	0.529	0.447	0.007	0.25	0.461	0.257	0.91	0.742
2375.179976	K.APNPPTFSELSHCRGAPELPR.E	*SHROOM2*	0.733	1	0.945	0.0009	0.164	0.25	0.304	0.203	0.195
2380.168494	x		0.168	0.359	0.547	0.021	0.944	0.8	0.003	0.014	0.008
2404.25209	K.LYLQDNAISHIPYNTLAKMR.E	*FLRT1*	0.955	0.301	0.148	0.708	1	0.945	0.042	0.008	0.016
2405.091621	K.LYLQDNAISHIPYNTLAKMR.E	*FLRT1*	0.104	0.57	0.844	0.256	0.933	0.675	0.006	0.295	0.295
2433.138151	R.KQYRPDMMSLQIQMYQLSR.L	*TBC1D4*	0.946	0.652	0.844	0.001	0.039	0.023	0.179	0.203	0.195
2434.139292	R.KQYRPDMMSLQIQMYQLSR.L	*TBC1D4*	0.733	0.25	0.383	0.005	0.164	0.039	0.288	0.359	0.313
2435.141433	R.KQYRPDMMSLQIQMYQLSR.L	*TBC1D4*	0.733	0.91	0.945	0.005	0.164	0.055	0.082	0.25	0.148
2588.064237	x		0.008	0.441	0.673	0.026	0.014	0.022	0.288	0.02	0.008

x- stands for peaks unidentified to peptides/proteins
Abbreviations: *TR – topographic region*.

Supplementary Table S6. The results of gene ontology (GO) term overrepresentation analysis. Analysis was conducted for GO category: Biological Process(Ge et al., 2020). Analysis embraced 25 proteins revealed as discriminative between eyes in patients with KTCN: paired model (see Supplementary Table S5). The GO term ID, GO term name, false discovery rate (FDR), number of genes associated with the GO term, fold enrichment, and the specific proteins from our dataset assigned to each GO term are indicated.

GO term	Enrichment FDR	nGenes	GO term Genes	Fold Enrichment	Genes
GO:0045176 apical protein localization	0.0386	2	15	127.1167	*SHROOM2, ACTB*
GO:1902176 negative reg. of oxidative stress-induced intrinsic apoptotic signaling pathway	0.0386	2	19	100.3553	*HSPB1 PPIA*
GO:0015874 norepinephrine transport	0.0411	2	23	82.9022	*ACTB FFAR3*
GO:0070527 platelet aggregation	0.0386	3	79	36.2041	*ACTB HSPB1 PPIA*
GO:1902414 protein localization to cell junction	0.0386	3	103	27.7682	*RELN ACTB HSPB1*
GO:0034109 homotypic cell-cell adhesion	0.0386	3	104	27.5012	*ACTB HSPB1 PPIA*
GO:2001243 negative reg. of intrinsic apoptotic signaling pathway	0.0386	3	108	26.4826	*HSPB1 HELLS PPIA*
GO:0030168 platelet activation	0.0411	3	143	20.0009	*ACTB HSPB1 PPIA*
GO:0032879 reg. of localization	0.0411	10	2945	3.2373	*RELN LAMB1 TBC1D4 CORIN GAPVD1 ACTB ANK2 HSPB1 PPIA FFAR3*
GO:0006810 transport	0.0411	13	4823	2.5697	*TRIM23 GOLGA4 ACTB TBC1D4 CORIN ANK2 SHROOM2 GAPVD1 RELN S100A6 HSPB1 PPIA FFAR3*

Supplementary S7. Discriminative proteins in the non-paired model of KTCN. Table shows results of analysis in three setting: I) ll KTCN patients (*n*=62) *vs* all control individuals (*n*=14), II) all females with KTCN (*n*=4) *vs* control females (*n*=8), and III) all males with KTCN (*n*=58) *vs* control males(*n*=6). For each *topographic region* (*central/middle/peripheral TR*) and each setting, analysis was performed separately.

Analysis input	*TR1*	*TR2*	*TR3*
all KTCN patients (*n*=62) *vs* all control individuals (*n*=14)	KRT12, CWC25, 5 SV, ISL2, ABCA13, CLU, PPIA, WDR62, MLL, ALDH3A1, TBC1D4, GAL3ST4, CLEC4F, BTN2A2, GAPDH, ZFP106, USP31, TRRAP, CORIN, SUMO2, FFAR3, SHROOM2, GOLGA4, ENO1, TMEM217, CIITA, FAR2, STAB1, NUCB1, SRRM2, FGF8, ABCC1, HSPB1	FAT3, PSMC3, KRT12, GAL3ST4, CLEC4F, GAPVD1, HSPB1, KRT7, KRT3, STAB1, BTN2A2, NUCB1, ABCC1, FFAR3, SRRM2, ENO1, WDR62, USP31, INSRR, ANK2, SLC25A23, TMEM217, FAR2, BPIFA2, FLRT1, PPIA, ISL2	BTN2A2, KRT12, TMEM217, CLU, HSPB1, ABCC1, ISL2, TBC1D4, 5 SV, FAM186B, CLEC4F, GAL3ST4, SUMO2, LTBP4, GAPVD1, CWC25, ACTB, ANXA2, SLC25A23, FAR2, LRRC27, FGF8, ABCA13, NUCB1, USP31, SARG, KRT3, PSMC3, BPIFA2, TSEN54, WDR62, FFAR3, INSRR, MLL, STAB1, FLRT1, ANK2, PPIA, CORIN, CIITA, ZFP106, KRT7, MIF
Females with KTCN (*n*=4) *vs* control females (*n*=8)	CLU, HELLS, TIAM1, ALDH3A1, PTPLB, S100A6,	ALDH3A1	TBC1D4
Males with KTCN (*n*=58) *vs* control males(*n*=6)	KRT3, ALDH3A1, KRT12, FLYWCH1, SRRM2, MASTL, KRT76, HSPB1, MRGBP, RELN, ANK2, PIDD, RTCD1, TRIM16, GPATCH1, FAR2, GSTP1, CIITA, PTPN1, ABCA13, CLEC4F, ENO1, BPIFA2, TMEM217, WDR66, LIFR, PMS2CL, STAB1, ANXA2, FLRT1, TKT, ABCC1, TSEN54, WDR62, CORIN, FGF8, CWC25, 5 SV, TRIM23, FFAR3, ZFP106, BTN2A2, RMI1, NUCB1,	SHROOM2, HSPB1, , TIAM1, PIDD, TRIM23, AKR1C2, GAL3ST4, FAT3, CLEC4F, KRT12, LAMB1, ENO1, FAM186B, INSRR, LRRC27, ABCA13, KRT3, GAPVD1, ACTB, KRT7, EDEM2, TMEM217, ANK2, SLC25A23, BTN2A2, FAR2, PSMC3, CCDC74A, PTPLB, CLU, ALDH3A1, FABP5, NUCB1, LGR4, MTERFD2, S100A6, SUMO2, SRRM2, HELLS, SLC8A1, GANAB, ABCC1, GSTP1, FLRT1,	ENO1, RELN, BTN2A2, RMI1, TMEM217, FAM186B, GAL3ST4, CLEC4F, KRT12, ISL2, ABCA13, CLU, GOLGA4, NUCB1, SUMO2, HSPB1, TSEN54, LTBP4, ACTB, DOCK8, ANXA2, KRT7, TBC1D4, FABP5, CIITA, KRT3, MLL, KRT76, ABCC1, SARG, 5 SV, SLC8A1, WDR62, SLC25A23, GAPVD1, FAR2, PTPLB, PSMC3, STAB1, USP31, FLRT1, FFAR3,

	LTBP4, KRT7, C15orf52, FABP5, ISL2, SHROOM2, LRRC27, SARG, FAM186B, MLL, GAL3ST4, GOLGA4, DOCK8, CLU, SUMO2, AKR1C2, CCDC74A, USP31, TRRAP	ISL2, FFAR3, ANXA2, GPATCH1, BPIFA2, WDR62, C15orf52, RELN, STAB1, TRIM16, LTBP4, NPHP1, TSEN54, PKM2, CIITA, MIF	ANK2, ZFP106, ALDH3A1, MTERFD2, CCDC74A, LIFR, CWC25, LRRC27, GSTP1, TIAM1, MIF, TRIM23, PPIA, GPATCH1, TRRAP, CORIN, TRIM16, FGF8, FAM102B, LAMB1, AKR1C2

Abbreviations: KTCN – keratoconus, *TR – topographic region, TR1 – central topographic region*, *TR2 – middle topographic region*, *TR3 – peripheral topographic region*.

Supplementary Table S8. The results of gene ontology (GO) term overrepresentation analysis. Analysis was conducted for GO category: Biological Process(Ge et al., 2020). Analysis embraced 86 proteins revealed as discriminative between patients with KTCN and control individuals: non-paired model (see Supplementary Table S7). The GO term ID, GO term name, false discovery rate (FDR), number of genes associated with the GO term, fold enrichment, and the specific proteins from our dataset assigned to each GO term are indicated.

GO term	Enrichment FDR	nGenes	GO term Genes	Fold Enrichment	Genes
GO:0010756 positive reg. of plasminogen activation	0.0292	2	8	74.2890	*ANXA2, ENO1*
GO:2000644 reg. of receptor catabolic proc.	0.0428	2	11	54.0283	*ANXA2, PTPN1*
GO:0043968 histone H2A acetylation	0.0142	3	23	38.7595	*ACTB, MRGBP, TRRAP*
GO:1903779 reg. of cardiac conduction	0.0144	3	26	34.2872	*ANK2, CORIN, SLC8A1*
GO:0006692 prostanoid metabolic proc.	0.0103	4	50	23.7725	*AKR1C2, GSTP1, MIF, FABP5*
GO:0006693 prostaglandin metabolic proc.	0.0103	4	50	23.7725	*AKR1C2, GSTP1, MIF, FABP5*
GO:1905168 positive reg. of double-strand break repair via homologous recombination	0.0301	3	41	21.7431	*ACTB, MRGBP, TRRAP*
GO:2001243 negative reg. of intrinsic apoptotic signaling pathway	0.0002	7	108	19.2601	*ENO1, MIF, HSPB1, HELLS, PTPN1, CLU, PPIA*
GO:0043967 histone H4 acetylation	0.0149	4	74	16.0625	*ACTB, MRGBP, TRRAP, TRIM16*
GO:0006690 icosanoid metabolic proc.	0.0144	5	129	11.5177	*AKR1C2, GSTP1, MIF, FABP5, ABCC1*
GO:0033559 unsaturated fatty acid metabolic proc.	0.0428	4	117	10.1592	*AKR1C2, GSTP1, MIF, FABP5*
GO:2001234 negative reg. of apoptotic signaling pathway	0.0015	8	250	9.5090	*ENO1, GSTP1, MIF, HSPB1, HELLS, PTPN1, CLU, PPIA*
GO:0032869 cellular response to insulin stimulus	0.0428	5	206	7.2125	*PKM, INSRR, TBC1D4, PTPN1, FFAR3*
GO:0097193 intrinsic apoptotic signaling pathway	0.0144	7	317	6.5618	*ENO1, MIF, HSPB1, HELLS, PTPN1, CLU, PPIA*
GO:0008544 epidermis development	0.0292	7	384	5.4169	*KRT7, KRT76, KRT3, KRT12, TRIM16, LGR4, FABP5*
GO:1902532 negative reg. of intracellular signal transduction	0.0149	9	566	4.7251	*ENO1, GSTP1, MIF, HSPB1, HELLS, BTN2A2, PTPN1, CLU, PPIA*
GO:0006897 endocytosis	0.0284	9	634	4.2183	*ANXA2, PTPN1, ACTB, ANK2, CLEC4F, GAPVD1, ABCA13, CLU, STAB1*
GO:0080135 reg. of cellular response to stress	0.0142	11	782	4.1799	*ACTB, MRGBP, TRRAP, ENO1, GSTP1, PTPN1, MIF, HSPB1, CLU, SLC25A23, PPIA*
GO:0045859 reg. of protein kinase activity	0.0428	9	702	3.8097	*GSTP1, PPIA, PTPN1, HSPB1, RELN, MIF, CLU, ACTB, SLC8A1*
GO:1901699 cellular response to nitrogen compound	0.0500	9	734	3.6436	*PKM, INSRR, ABCC1, TBC1D4, CIITA, SLC8A1, PTPN1, ACTB, FFAR3*

GO:0043549 reg. of kinase activity	0.0347	10	826	3.5975	*INSRR, GSTP1, PPIA, PTPN1, HSPB1, RELN, MIF, CLU, ACTB, SLC8A1*
GO:0051338 reg. of transferase activity	0.0299	11	956	3.4192	*INSRR, GSTP1, PPIA, PTPN1, HSPB1, MASTL, RELN, MIF, CLU, ACTB, SLC8A1*
GO:0051240 positive reg. of multicellular organismal proc.	0.0015	19	1687	3.3467	*TIAM1, ACTB, GAPDH, CLU, GOLGA4, ANXA2, TRIM16, MIF, WDR62, HSPB1, FGF8, BTN2A2, FABP5, RELN, LGR4, ENO1, PKM, SLC8A1, FFAR3*
GO:0018193 peptidyl-amino acid modification	0.0215	14	1375	3.0256	*MRGBP, MASTL, SUMO2, PPIA, ACTB, TRRAP, PTPN1, TRIM16, MIF, INSRR, PKM, FGF8, RELN, GAPDH*
GO:0080134 reg. of response to stress	0.0403	14	1529	2.7209	*ACTB, MRGBP, TRRAP, ENO1, GSTP1, PTPN1, MIF, ABCC1, HSPB1, ANXA2, CLU, SLC25A23, PPIA, FFAR3*
GO:1901700 response to oxygen-containing compound	0.0428	15	1752	2.5441	*PKM, EDEM2, AKR1C2, SLC8A1, FFAR3, TRIM16, INSRR, ABCC1, TBC1D4, RMI1, PTPN1, MIF, ACTB, CLU, GSTP1*
GO:0051049 reg. of transport	0.0347	16	1874	2.5371	*ANXA2, ABCA13, PTPN1, EDEM2, TBC1D4, CORIN, FABP5, GAPVD1, SLC8A1, RELN, MIF, ACTB, CLU, ANK2, PPIA, FFAR3*
GO:0016310 phosphorylation	0.0284	17	1994	2.5334	*INSRR, PKM, ENO1, FGF8, GAPDH, MASTL, GSTP1, PPIA, PTPN1, MIF, HSPB1, CIITA, RELN, TRRAP, CLU, ACTB, SLC8A1*
GO:0071310 cellular response to organic substance	0.0144	21	2609	2.3918	*PKM, FGF8, LIFR, LGR4, GSTP1, GAPDH, AKR1C2, FFAR3, PTPN1, INSRR, LTBP4, ABCC1, HELLS, TBC1D4, CIITA, SLC8A1, MIF, ACTB, HSPB1, DOCK8, CLU*
GO:0048584 positive reg. of response to stimulus	0.0299	19	2427	2.3263	*BTN2A2, ACTB, LAMB1, MRGBP, GAPDH, AKR1C2, PPIA, TRRAP, LGR4, TRIM16, MIF, ABCC1, FGF8, CLU, FABP5, RELN, PTPN1, HSPB1, FFAR3*
GO:0032879 reg. of localization	0.0143	23	2945	2.3207	*DOCK8, FGF8, ANXA2, RELN, LAMB1, TIAM1, ABCA13, PTPN1, MIF, WDR62, EDEM2, SLC25A23, TBC1D4, CORIN, FABP5, GAPVD1, SLC8A1, ACTB, CLU, ANK2, HSPB1, PPIA, FFAR3*

GO:0050790 reg. of catalytic activity	0.0284	20	2582	2.3017	*INSRR, DOCK8, TBC1D4, TIAM1, LGR4, GSTP1, GAPDH, PPIA, PTPN1, NUCB1, HSPB1, TRIM23, MASTL, GAPVD1, ANXA2, RELN, MIF, CLU, ACTB, SLC8A1*
GO:0010033 response to organic substance	0.0142	25	3269	2.2725	*PKM, EDEM2, FGF8, LIFR, LGR4, GSTP1, GAPDH, CLU, AKR1C2, CIITA, FFAR3, PTPN1, TRIM16, INSRR, LTBP4, ABCC1, HELLS, TBC1D4, ANK2, RMI1, SLC8A1, MIF, ACTB, HSPB1, DOCK8*
GO:0051239 reg. of multicellular organismal proc.	0.0144	23	3024	2.2601	*BTN2A2, TIAM1, ACTB, SLC8A1, GSTP1, GAPDH, CLU, GOLGA4, ANXA2, TRIM16, MIF, WDR62, HSPB1, FGF8, CORIN, FABP5, RELN, LGR4, ENO1, ANK2, STAB1, PKM, FFAR3*
GO:0070887 cellular response to chemical stimulus	0.0142	25	3300	2.2512	*PKM, FGF8, LIFR, LGR4, GSTP1, ENO1, GAPDH, AKR1C2, SLC8A1, FFAR3, PPIA, PTPN1, MIF, INSRR, LTBP4, ABCC1, HSPB1, HELLS, SLC25A23, TBC1D4, CIITA, ACTB, DOCK8, CLU, ALDH3A1*
GO:0023051 reg. of signaling	0.0248	25	3615	2.0550	*GSTP1, LAMB1, RELN, ENO1, GAPDH, AKR1C2, ANXA2, PPIA, PTPN1, LGR4, TRIM16, MIF, HSPB1, FGF8, HELLS, BTN2A2, TIAM1, FABP5, CLU, ANK2, FFAR3, LTBP4, DOCK8, CORIN, SLC8A1*
GO:0042221 response to chemical	0.0144	31	4821	1.9108	*PKM, EDEM2, FGF8, LIFR, LGR4, GSTP1, ENO1, GAPDH, CLU, AKR1C2, CIITA, SLC8A1, FFAR3, PPIA, PTPN1, TRIM16, MIF, INSRR, LTBP4, ABCC1, HSPB1, HELLS, SLC25A23, TBC1D4, ANK2, ISL2, RMI1, RELN, ACTB, DOCK8, ALDH3A1*
GO:0048583 reg. of response to stimulus	0.0428	27	4338	1.8495	*BTN2A2, ACTB, GSTP1, LAMB1, MRGBP, TRRAP, ENO1, GAPDH, AKR1C2, PPIA, PTPN1, LGR4, TRIM16, MIF, ABCC1, HSPB1, FGF8, HELLS, CLU, TIAM1, FABP5, ANXA2, RELN, SLC25A23, FFAR3, LTBP4, DOCK8*

Supplementary Table S9.The detailed clinical data of examined KTCN from the rediscovery group.
Clinical data concerning both eyes is presented. The eye with more advanced disease is indicated. Samples subjected to RT-qPCR or immunofluorescence stainings (IF) experiments are annotated.

Patient ID	Diagnosis	Sex	Age	Examined eye	More advanced eye	K1 [D]	K2 [D]	Kmax [D]	Anterior Elevation [µm]	Posterior Elevation [µm]	TCT [µm]	TKC Grade	TET (in range 0.0-7.0mm) [µm]	Average thickness of *TR1* [µm]	Average thickness of *TR2* [µm]	Average thickness of *TR3* [µm]	Samples subjected to RT-qPCR	Samples subjected to IF
66 KTCN	KTCN	M	20	OS	yes	43.8	48.1	54.5	29	31	467	2	43	47	56	51	yes	no
				OD	no	43	44.6	45.6	8	9	489	1	46	51	55	49	yes	no
79 KTCN	KTCN	F	18	OS	yes	51.9	58.4	70.8	59	106	454	3-4	43	46	67	53	yes	no
				OD	no	41.7	43.1	43.9	3	18	530	1-2	47	47	50	50	yes	no
97 KTCN	KTCN	M	19	OS	no	41.3	42.9	46.1	13	29	508	1	53	54	60	55	no	yes
				OD	yes	42.0	45.6	51.0	17	36	500	2	40	44	55	51	no	yes
103 KTCN	KTCN	F	13	OS	yes	46.6	47.7	55.2	29	68	515	2-3	36	40	55	49	yes	no
				OD	no	44	44.8	45.6	6	16	557	FF	39	44	48	46	yes	no
133 KTCN	KTCN	M	24	OS	no	43.5	45.7	50.7	24	52	430	2	45	48	61	54	yes	no
				OD	yes	53.4	59.1	74.8	80	136	364	4	41	46	70	59	yes	no
154 KTCN	KTCN	M	27	OS	no	41.4	42.3	43.4	6	12	469	FF	50	52	56	51	yes	no
				OD	yes	42.1	44.1	48.3	14	37	452	1-2	50	51	60	50	yes	no
161 KTCN	KTCN	M	22	OS	yes	53.6	53.9	70.3	53	94	449	3-4	49	51	65	53	yes	no
				OD	no	44.8	45.4	50	14	29	503	1-1	45	49	61	51	yes	no
162 KTCN	KTCN	M	23	OS	yes	43.7	45.4	51.9	22	47	459	2	40	45	57	50	no	yes
				OD	no	41.8	43.6	43.9	4	14	486	FF	46	51	54	49	no	yes
166 KTCN	KTCN	M	23	OS	yes	53.6	59.8	71.4	43	81	411	3	40	45	59	52	yes	no
				OD	no	44	47	51.4	15	43	470	1-2	47	49	57	52	yes	no
184 KTCN	KTCN	M	18	OS	yes	44.5	50.1	55.1	34	54	520	3	42	47	60	60	yes	no
				OD	no	42.6	44.4	45.6	9	25	557	1	50	52	56	50	yes	no
189 KTCN	KTCN	M	26	OS	yes	55.3	66.8	79.4	61	87	440	3-4	33	40	68	54	yes	no
				OD	no	45.3	46.5	47.5	8	21	535	FF	52	54	61	54	yes	no
194 KTCN	KTCN	M	16	OS	no	43	43	44.9	7	18	500	FF	50	58	63	53	yes	no
				OD	yes	45.6	47.2	55	28	62	454	2	44	48	62	53	yes	no
196 KTCN	KTCN	M	30	OS	yes	46.4	49.1	68	54	97	431	3-4	43	45	65	49	yes	no

				OD	no	42	42	46.8	11	27	502	1	51	51	63	49	yes	no
198 KTCN	KTCN	M	18	OS	no	42.0	42.9	43.4	3	12	532	FF	51	56	58	52	no	yes
				OD	yes	46.0	48.4	64.1	29	69	459	3	50	52	60	58	no	yes
201 KTCN	KTCN	M	19	OS	no	42.1	43.4	43.5	3	11	520	FF	48	57	52	48	yes	no
				OD	yes	43.2	45	48.6	14	31	501	1-2	47	49	58	50	yes	no
202 KTCN	KTCN	M	21	OS	no	41.6	42.5	43.0	6	16	525	FF	47	49	51	48	no	yes
				OD	yes	42.0	42.8	43.4	7	15	521	1	47	48	51	48	no	yes
206 KTCN	KTCN	M	14	OS	yes	56.6	58.4	69.7	69	124	505	4	46	50	63	55	no	yes
				OD	no	40.3	41.5	46.0	14	23	535	1	52	53	59	53	no	yes
218 KTCN	KTCN	M	16	OS	yes	47.3	52	59.4	34	69	472	3	45	49	58	50	yes	no
				OD	no	44.9	46.7	50.2	13	36	500	1	48	50	57	51	yes	no
221 KTCN	KTCN	F	23	OS	yes	43.8	46.1	49.3	9	26	474	1	44	48	61	58	no	yes
				OD	no	42.6	43.5	43.9	5	13	503	FF	52	54	59	57	no	yes
225 KTCN	KTCN	M	29	OS	no	42.5	42.8	46.7	9	31	446	1	52	54	60	58	no	yes
				OD	yes	44.3	45.4	52.9	23	48	418	2	43	49	62	60	no	yes

Abbreviations in table: D – diopters, F – female, FF – forme fruste, K1 – flat keratometry, K2 – steep keratometry, Kmax – maximal corneal curvature, KTCN- keratoconus, M – male, OD – right eye, OS – left eye, TCT – thinnest corneal thickness, TET – thinnest epithelial thickness, TKC – Topographic Keratoconus Classification, *TR1 – central topographic region*, *TR2 – middle topographic region*, *TR3 – peripheral topographic region.*.

Supplementary Table S10. The RT-qPCR data used for the validation of study results. The Ct values obtained towards validation of the relative expression of *TFRC*, *TLR2* and *MSMB* genes in each of 78 additional CE experimental samples (both eyes of 13 KTCN patients with designated three *topographic regions*) and three CE samples included in MALDI-TOF/TOF MS but not RNA-Seq experiments (samples: 146 OPT/E/OS/1, 146/OPT/E/OS/2, and 146 OPT/E/OS/3). The expression of *LDHA*, *RLP4*, and *UBC* genes was used for data normalization. Each reaction was performed in triplicates, and if one of the replicates deviated by ≥0.5 Ct it was removed.

Experimental samples ID	*LDHA*	*LDHA*	*LDHA*	*UBC*	*UBC*	*UBC*	*RPL4*	*RPL4*	*RPL4*	*TFRC*	*TFRC*	*TFRC*	*TLR2*	*TLR2*	*TLR2*	*MSMB*	*MSMB*	*MSMB*
66 KTCN/OD/1	21.54	21.30	21.55	21.21	21.06	20.94	20.35	20.38	20.42	23.03	23.04	23.00	-	-	-	-	-	-
66 KTCN/OS/1	21.07	21.19	21.20	20.32	20.29	20.21	20.58	20.41	20.13	22.40	22.39	22.27	-	-	-	-	-	-
79 KTCN/OD/1	21.11	21.11	21.03	20.52	20.39	20.33	20.09	20.06	19.74	22.36	22.53	22.28	-	-	-	-	-	-
79 KTCN/OS/1	21.02	21.02	21.17	20.32	20.34	20.37	20.49	20.73	20.32	21.10	-	21.45	-	-	-	-	-	-
103 KTCN/OD/1	20.79	20.75	21.08	20.27	20.15	20.17	20.20	20.40	20.01	21.81	22.10	21.80	-	-	-	-	-	-
103 KTCN/OS/1	22.72	22.90	22.83	21.31	21.29	21.33	22.06	-	21.75	23.55	23.62	23.40	-	-	-	-	-	-
133 KTCN/OD/1	21.28	21.66	21.55	20.42	20.39	20.49	20.87	20.47	20.52	22.56	22.32	22.40	-	-	-	-	-	-
133 KTCN/OS/1	20.83	-	21.15	20.38	20.44	20.31	20.39	20.61	20.37	21.70	-	21.69	-	-	-	-	-	-
154 KTCN/OD/1	22.27	22.18	-	21.09	21.25	20.92	-	21.91	21.94	22.33	22.40	-	-	-	-	-	-	-
154 KTCN/OS/1	21.79	21.76	-	20.27	20.42	20.70	20.62	20.88	-	22.19	22.24	-	-	-	-	-	-	-
161 KTCN/OD/1	21.60	21.52	-	20.73	20.94	20.73	-	21.34	21.32	22.16	22.16	-	-	-	-	-	-	-
161 KTCN/OS/1	21.98	22.09	-	20.63	20.87	20.87	22.42	22.61	-	22.93	23.12	22.67	-	-	-	-	-	-
166 KTCN/OD/1	20.71	20.76	20.61	20.23	20.45	20.36	20.73	-	21.14	21.94	22.00	21.97	-	-	-	-	-	-
166 KTCN/OS/1	20.84	20.98	20.69	20.94	20.57	20.92	20.31	20.50	-	22.06	22.20	-	-	-	-	-	-	-
184 KTCN/OD/1	21.21	21.44	-	20.61	21.06	20.80	22.24	22.00	-	22.42	22.52	-	-	-	-	-	-	-
184 KTCN/OS/1	20.61	20.55	-	20.46	20.49	20.23	20.23	20.42	-	21.44	21.64	-	-	-	-	-	-	-
189 KTCN/OD/1	20.50	20.63	-	21.03	21.00	21.11	21.52	21.30	21.67	21.59	21.93	-	-	-	-	-	-	-
189 KTCN/OS/1	-	21.53	21.62	20.97	21.26	21.19	21.67	22.02	22.09	-	22.45	22.38	-	-	-	-	-	-
194 KTCN/OD/1	-	21.12	21.10	20.61	20.78	20.90	21.41	21.40	21.59	21.48	21.75	21.79	-	-	-	-	-	-
194 KTCN/OS/1	20.67	21.11	21.13	20.69	20.76	20.62	21.41	21.64	21.81	-	22.36	22.29	-	-	-	-	-	-
196 KTCN/OD/1	-	21.10	20.89	21.22	21.11	21.17	21.09	21.03	-	-	23.19	23.05	-	-	-	-	-	-
196 KTCN/OS/1	20.77	21.07	20.92	21.15	21.03	20.72	21.20	21.43	21.56	-	21.84	21.76	-	-	-	-	-	-
201 KTCN/OD/1	20.57	20.77	20.81	20.46	20.33	20.19	21.07	21.21	21.30	21.74	22.22	21.81	-	-	-	-	-	-
201 KTCN/OS/1	-	20.64	20.82	20.51	20.27	20.36	21.15	21.04	21.08	21.86	-	21.80	-	-	-	-	-	-
218 KTCN/OD/1	22.15	21.85	-	21.04	20.90	20.80	22.24	22.08	-	23.55	23.46	23.39	-	-	-	-	-	-
218 KTCN/OS/1	21.81	21.59	21.84	21.37	21.34	20.91	21.69	21.91	-	22.71	23.07	22.89	-	-	-	-	-	-

66 KTCN/OD/2	20.44	20.71	-	20.68	21.06	20.87	21.72	22.12	21.64	23.15	23.20	23.16	-	-	-	-	24.62	24.41
66 KTCN/OS/2	-	20.67	20.77	20.32	20.26	20.39	21.20	21.44	21.24	22.47	-	22.36	-	-	-	24.50	24.53	24.19
79 KTCN/OD/2	20.65	21.02	20.78	20.37	20.50	20.39	-	21.87	21.56	23.22	-	23.08	-	-	-	24.10	24.11	23.73
79 KTCN/OS/2	20.24	20.54	-	20.61	20.38	20.40	21.44	21.76	21.60	-	21.79	21.69	-	-	-	22.69	22.74	22.63
103 KTCN/OD/2	-	20.35	20.62	20.51	20.65	20.28	21.18	21.40	21.31	22.24	22.15	22.58	-	-	-	26.56	26.32	26.23
103 KTCN/OS/2	-	20.70	20.58	20.73	20.31	20.48	21.32	21.20	21.34	-	21.73	21.78	-	-	-	26.07	26.34	26.18
133 KTCN/OD/2	19.78	-	20.01	20.37	20.21	20.28	21.03	21.03	20.88	21.83	21.33	21.37	-	-	-	24.44	24.50	24.25
133 KTCN/OS/2	20.16	20.39	20.33	20.45	20.57	20.37	21.26	21.36	21.30	21.73	21.70	21.57	-	-	-	25.33	25.25	25.17
154 KTCN/OD/2	21.14	20.94	21.11	20.77	20.70	20.68	21.65	21.61	-	22.78	22.55	-	-	-	-	24.32	24.41	-
154 KTCN/OS/2	20.56	20.31	20.47	20.35	20.76	20.29	21.22	21.39	-	22.10	22.00	-	-	-	-	24.71	24.69	-
161 KTCN/OD/2	20.96	20.62	20.82	20.56	20.88	20.59	21.45	21.49	-	22.44	22.26	-	-	-	-	25.05	24.99	-
161 KTCN/OS/2	21.25	21.32	21.47	21.08	21.04	21.08	21.77	22.01	-	23.10	23.17	-	-	-	-	25.76	25.93	-
166 KTCN/OD/2	20.12	20.31	20.35	20.60	-	20.56	21.33	21.08	-	22.65	22.58	22.26	-	-	-	26.47	26.55	-
166 KTCN/OS/2	20.48	20.60	20.30	20.87	20.97	-	21.63	21.46	-	21.82	21.89	-	-	-	-	26.30	26.23	-
184 KTCN/OD/2	20.23	20.56	20.59	20.79	21.03	-	21.36	21.25	-	22.07	22.26	-	-	-	-	24.88	24.76	-
184 KTCN/OS/2	20.21	20.23	20.35	20.94	20.88	20.61	-	19.98	19.52	-	19.42	19.68	-	-	-	25.68	25.63	-
189 KTCN/OD/2	21.37	21.18	21.00	20.83	21.00	20.97	-	21.32	21.57	22.20	22.40	22.48	-	-	-	-	26.29	26.40
189 KTCN/OS/2	20.65	20.51	20.59	20.19	20.60	20.50	-	20.62	21.10	21.47	21.30	-	-	-	-	25.20	-	25.35
194 KTCN/OD/2	21.15	21.11	21.05	20.83	20.66	20.93	-	21.19	21.43	21.72	21.90	21.78	-	-	-	24.20	-	24.42
194 KTCN/OS/2	20.46	-	20.61	20.54	20.42	20.36	-	20.54	20.97	21.66	21.58	21.52	-	-	-	25.27	25.35	25.64
196 KTCN/OD/2	20.46	20.82	20.45	20.39	20.30	20.67	-	20.19	20.64	21.92	22.06	22.19	-	-	-	22.68	22.39	22.42
196 KTCN/OS/2	20.44	20.31	20.41	20.43	20.48	20.75	20.45	20.81	20.88	21.94	21.75	21.85	-	-	-	22.20	22.19	22.60
201 KTCN/OD/2	20.40	20.30	20.32	20.25	20.11	20.08	-	20.94	20.85	-	21.76	21.94	-	-	-	24.69	24.66	-
201 KTCN/OS/2	20.33	20.42	20.29	20.22	20.24	20.29	-	21.01	20.86	22.53	22.52	-	-	-	-	-	24.59	24.66
218 KTCN/OD/2	20.93	21.01	20.77	20.74	20.77	20.42	-	21.15	20.90	22.88	22.88	22.42	-	-	-	22.94	22.97	22.64
218 KTCN/OS/2	21.06	20.77	20.66	20.57	20.49	20.76	20.88	20.66	-	22.33	22.33	22.35	-	-	-	22.92	22.81	22.92
66 KTCN/OD/3	21.24	21.60	21.58	21.48	21.17	21.64	21.67	21.91	21.78	22.72	22.64	22.46	28.78	28.47	28.87	-	-	-
66 KTCN/OS/3	20.90	21.05	20.73	20.60	20.21	20.53	20.94	21.04	21.04	-	22.09	21.86	27.57	27.53	27.66	-	-	-
79 KTCN/OD/3	21.13	21.30	21.11	20.86	20.78	21.04	21.21	21.34	21.12	23.00	22.59	22.51	28.32	28.18	28.29	-	-	-
79 KTCN/OS/3	21.08	21.35	21.02	20.45	20.81	20.78	21.46	21.51	21.50	22.60	22.19	22.23	27.51	27.58	27.59	-	-	-
103 KTCN/OD/3	21.60	21.99	21.74	21.37	21.14	21.41	21.87	21.79	21.60	23.46	23.12	23.04	29.10	28.76	29.14	-	-	-
103 KTCN/OS/3	21.17	21.34	21.07	20.78	-	20.59	21.31	21.19	21.15	-	22.29	22.04	28.42	28.60	28.54	-	-	-

133 KTCN/OD/3	21.00	20.97	21.05	21.19	20.78	20.81	20.99	20.83	21.01	21.74	21.37	21.27	27.77	27.79	27.76	-	-	-
133 KTCN/OS/3	20.37	20.78	20.53	20.84	-	20.75	20.89	20.81	20.87	22.27	22.16	22.07	27.71	27.72	27.94	-	-	-
154 KTCN/OD/3	21.63	21.19	21.46	21.16	21.23	-	21.33	21.20	-	22.48	22.61	22.29	28.55	28.15	-	-	-	-
154 KTCN/OS/3	20.92	20.64	21.06	20.72	21.02	-	20.84	20.97	-	22.17	21.92	21.95	28.07	28.26	27.92	-	-	-
161 KTCN/OD/3	21.12	21.10	21.28	21.13	21.15	-	21.31	21.21	-	22.95	22.79	22.79	29.40	29.12	29.04	-	-	-
161 KTCN/OS/3	21.34	21.10	-	21.21	21.31	-	21.33	21.45	-	23.05	22.88	23.14	29.46	29.40	29.04	-	-	-
166 KTCN/OD/3	20.40	20.22	20.38	20.88	21.00	-	20.94	21.10	-	21.99	21.72	21.89	-	27.44	27.18	-	-	-
166 KTCN/OS/3	20.88	20.84	20.61	20.87	20.71	-	21.23	21.06	-	21.74	21.81	21.82	27.84	27.97	-	-	-	-
184 KTCN/OD/3	20.87	20.92	20.73	21.19	21.11	-	20.95	21.08	-	21.75	21.92	21.86	28.35	-	28.04	-	-	-
184 KTCN/OS/3	20.64	20.88	20.73	21.12	21.10	-	20.78	20.96	-	21.15	21.07	21.15	28.57	28.34	28.45	-	-	-
189 KTCN/OD/3	20.68	-	20.50	-	20.48	20.80	20.35	20.44	20.27	21.41	21.82	21.89	-	28.54	28.20	-	-	-
189 KTCN/OS/3	20.20	20.02	20.29	20.06	20.28	-	20.52	20.15	20.37	21.71	21.74	21.63	27.84	27.94	27.85	-	-	-
194 KTCN/OD/3	-	20.07	20.32	19.86	20.35	-	20.80	20.84	21.24	21.89	21.76	21.80	27.78	28.04	-	-	-	-
194 KTCN/OS/3	19.90	20.01	-	20.12	-	20.06	20.82	-	20.96	21.58	21.57	21.65	-	28.24	28.00	-	-	-
196 KTCN/OD/3	-	20.06	20.32	20.01	20.35	-	20.24	20.30	-	21.78	21.90	21.88	28.12	28.30	28.31	-	-	-
196 KTCN/OS/3	-	20.43	20.41	20.74	20.43	20.53	20.50	20.55	20.82	21.86	21.95	21.65	28.07	27.75	28.21	-	-	-
201 KTCN/OD/3	19.86	20.07	-	20.08	19.60	-	20.58	20.76	20.97	22.39	22.28	22.31	27.84	27.66	27.69	-	-	-
201 KTCN/OS/3	19.65	20.02	-	20.07	20.54	20.41	20.36	20.10	-	21.47	21.46	21.40	27.69	28.02	27.92	-	-	-
218 KTCN/OD/3	-	20.33	20.29	21.79	21.35	-	21.18	20.96	21.22	22.15	22.22	-	-	28.24	28.45	-	-	-
218 KTCN/OS/3	20.45	20.27	20.52	21.06	20.82	-	-	20.81	20.73	22.12	22.19	22.28	28.52	28.56	28.74	-	-	-

Supplementary Table S11. Quantitative analysis of TFRC (*blue*), FTL (*green*), and FTH1 (*yellow*) protein expression in CE samples of a patient with KTCN. Fluorescence intensities were quantified for the *topographic region* of CE samples from seven KTCN patients using Fiji (ImageJ)(Schindelin et al., 2012). Measurements were based on median fluorescence values normalized to the nuclear signal from propidium iodide (PI; *red*) staining. For each patient, data are provided for both the less and more affected eyes. The table includes: *topographic region*, fluorescence channel (ch00: *blue*, ch01: *yellow*, ch02: *red*, ch03: *green*), region of interest (ROI), mean, maximum, and median fluorescence intensity, as well as normalized median fluorescence intensity for each eye.

ID short	More advanced eye	region	channel	ROI	Mean intensity of more advanced eye	Max intensity of more advanced eye	Median intensity of more advanced eye	Normalized median intensity of more advanced eye	Mean intensity of less advanced eye	Max intensity of less advanced eye	Median intensity of less advanced eye	Normalized median intensity of more advanced eye
97 KTCN	OD	*TR2*	ch00	39.278	14.248	41	14	0.875	13.769	41	13	0.282609
97 KTCN	OD	*TR2*	ch01	39.278	6.055	58	6	0.375	13.627	170	13	0.282609
97 KTCN	OD	*TR2*	ch02	39.278	15.855	42	16	1	44.333	85	46	1
97 KTCN	OD	*TR2*	ch03	39.278	3.615	47	3	0.1875	7.282	31	7	0.152174
97 KTCN	OD	*TR3*	ch00	39.278	16.431	51	16	0.4	15.601	57	15	0.220588
97 KTCN	OD	*TR3*	ch01	39.278	13.093	84	13	0.325	15.477	109	15	0.220588
97 KTCN	OD	*TR3*	ch02	39.278	39.050	85	40	1	63.035	85	68	1
97 KTCN	OD	*TR3*	ch03	39.278	6.792	28	7	0.175	6.598	85	6	0.088235
162 KTCN	OS	*TR1*	ch00	39.278	6.627	25	6	0.272727	6.921	28	7	0.636364
162 KTCN	OS	*TR1*	ch01	39.278	8.820	33	9	0.409091	8.394	48	8	0.727273
162 KTCN	OS	*TR1*	ch02	39.278	21.579	66	22	1	11.831	53	11	1
162 KTCN	OS	*TR1*	ch03	39.278	3.792	14	4	0.181818	3.204	27	3	0.272727
162 KTCN	OS	*TR2*	ch00	39.278	11.430	37	11	0.23913	9.835	40	10	0.333333
162 KTCN	OS	*TR2*	ch01	39.278	13.406	125	13	0.282609	11.798	170	11	0.366667
162 KTCN	OS	*TR2*	ch02	39.278	43.568	85	46	1	29.292	85	30	1
162 KTCN	OS	*TR2*	ch03	39.278	5.644	24	5	0.108696	4.853	21	5	0.166667
162 KTCN	OS	*TR3*	ch00	39.278	14.033	42	13	0.270833	8.651	30	8	0.210526
162 KTCN	OS	*TR3*	ch01	39.278	15.700	55	15	0.3125	13.841	170	13	0.342105
162 KTCN	OS	*TR3*	ch02	39.278	46.237	85	48	1	38.603	85	38	1
162 KTCN	OS	*TR3*	ch03	39.278	8.369	37	8	0.166667	5.372	22	5	0.131579
198 KTCN	OD	*TR1*	ch00	39.278	12.078	45	11	0.354839	11.767	44	11	0.323529
198 KTCN	OD	*TR1*	ch01	39.278	10.773	115	10	0.322581	12.995	111	13	0.382353

198 KTCN	OD	*TR1*	ch02	39.278	28.275	71	31	1	33.638	85	34	1
198 KTCN	OD	*TR1*	ch03	39.278	2.825	35	3	0.096774	4.576	40	4	0.117647
198 KTCN	OD	*TR2*	ch00	39.278	17.870	58	17	0.242857	11.606	38	11	0.139241
198 KTCN	OD	*TR2*	ch01	39.278	17.138	114	17	0.242857	21.388	72	21	0.265823
198 KTCN	OD	*TR2*	ch02	39.278	56.751	85	70	1	70.677	85	79	1
198 KTCN	OD	*TR2*	ch03	39.278	5.704	46	5	0.071429	8.778	32	9	0.113924
198 KTCN	OD	*TR3*	ch00	39.278	8.013	29	8	0.266667	21.223	65	21	0.259259
198 KTCN	OD	*TR3*	ch01	39.278	11.263	70	11	0.366667	19.163	117	19	0.234568
198 KTCN	OD	*TR3*	ch02	39.278	28.321	85	30	1	66.594	85	81	1
198 KTCN	OD	*TR3*	ch03	39.278	3.893	36	4	0.133333	6.628	36	6	0.074074
202 KTCN	OD	*TR1*	ch00	39.278	5.996	26	6	0.136364	9.885	35	10	0.2
202 KTCN	OD	*TR1*	ch01	39.278	11.061	170	11	0.25	12.193	87	12	0.24
202 KTCN	OD	*TR1*	ch02	39.278	43.662	85	44	1	45.370	85	50	1
202 KTCN	OD	*TR1*	ch03	39.278	4.620	34	4	0.090909	5.826	42	5	0.1
202 KTCN	OD	*TR2*	ch00	39.278	9.287	37	9	0.15	11.072	34	11	0.146667
202 KTCN	OD	*TR2*	ch01	39.278	12.910	170	13	0.216667	14.772	109	15	0.2
202 KTCN	OD	*TR2*	ch02	39.278	56.947	85	60	1	66.798	85	75	1
202 KTCN	OD	*TR2*	ch03	39.278	5.148	47	5	0.083333	5.112	27	5	0.066667
202 KTCN	OD	*TR3*	ch00	39.278	13.345	54	13	0.168831	8.839	29	9	0.191489
202 KTCN	OD	*TR3*	ch01	39.278	14.901	134	15	0.194805	11.070	37	11	0.234043
202 KTCN	OD	*TR3*	ch02	39.278	66.119	85	77	1	45.443	85	47	1
202 KTCN	OD	*TR3*	ch03	39.278	5.233	38	5	0.064935	6.201	18	6	0.12766
206 KTCN	OS	*TR1*	ch00	39.278	14.729	41	14	0.4375	11.236	39	11	0.392857
206 KTCN	OS	*TR1*	ch01	39.278	16.141	170	15	0.46875	10.835	170	11	0.392857
206 KTCN	OS	*TR1*	ch02	39.278	30.807	84	32	1	26.301	71	28	1
206 KTCN	OS	*TR1*	ch03	39.278	11.288	85	11	0.34375	4.972	49	5	0.178571
206 KTCN	OS	*TR2*	ch00	39.278	14.712	53	14	0.538462	17.861	54	17	0.320755
206 KTCN	OS	*TR2*	ch01	39.278	15.235	170	14	0.538462	14.556	49	14	0.264151
206 KTCN	OS	*TR2*	ch02	39.278	25.993	85	26	1	51.444	85	53	1
206 KTCN	OS	*TR2*	ch03	39.278	10.683	85	10	0.384615	6.526	28	6	0.113208
206 KTCN	OS	*TR3*	ch00	39.278	19.894	60	19	0.311475	20.549	68	20	0.30303
206 KTCN	OS	*TR3*	ch01	39.278	18.929	71	19	0.311475	17.958	155	18	0.272727

206 KTCN	OS	*TR3*	ch02	39.278	55.522	85	61	1	59.470	85	66	1
206 KTCN	OS	*TR3*	ch03	39.278	11.746	35	11	0.180328	9.777	85	10	0.151515
221 KTCN	OS	*TR1*	ch00	39.278	7.811	30	7	0.212121	9.699	33	9	0.225
221 KTCN	OS	*TR1*	ch01	39.278	9.074	107	9	0.272727	11.202	61	11	0.275
221 KTCN	OS	*TR1*	ch02	39.278	31.829	85	33	1	37.683	85	40	1
221 KTCN	OS	*TR1*	ch03	39.278	4.240	37	4	0.121212	7.406	32	7	0.175
221 KTCN	OS	*TR2*	ch00	39.278	14.433	50	14	0.259259	8.272	85	8	0.145455
221 KTCN	OS	*TR2*	ch01	39.278	12.455	170	12	0.222222	13.894	149	13	0.236364
221 KTCN	OS	*TR2*	ch02	39.278	51.171	85	54	1	53.184	85	55	1
221 KTCN	OS	*TR2*	ch03	39.278	4.763	85	4	0.074074	7.158	40	7	0.127273
221 KTCN	OS	*TR3*	ch00	39.278	9.717	33	9	0.126761	9.252	33	9	0.128571
221 KTCN	OS	*TR3*	ch01	39.278	14.682	95	15	0.211268	15.389	170	15	0.214286
221 KTCN	OS	*TR3*	ch02	39.278	62.406	85	71	1	63.710	85	70	1
221 KTCN	OS	*TR3*	ch03	39.278	8.480	57	8	0.112676	7.181	49	7	0.1
225 KTCN	OD	*TR1*	ch00	39.278	6.674	22	6	0.230769	3.652	16	3	0.230769
225 KTCN	OD	*TR1*	ch01	39.278	10.524	65	10	0.384615	12.208	72	11	0.846154
225 KTCN	OD	*TR1*	ch02	39.278	24.645	77	26	1	13.163	37	13	1
225 KTCN	OD	*TR1*	ch03	39.278	2.642	30	2	0.076923	1.645	13	2	0.153846
225 KTCN	OD	*TR2*	ch00	39.278	7.976	32	8	0.32	5.820	22	6	0.285714
225 KTCN	OD	*TR2*	ch01	39.278	9.969	65	9	0.36	9.960	170	9	0.428571
225 KTCN	OD	*TR2*	ch02	39.278	25.115	70	25	1	23.815	85	21	1
225 KTCN	OD	*TR2*	ch03	39.278	6.309	69	5	0.2	2.296	16	2	0.095238
225 KTCN	OD	*TR3*	ch00	39.278	10.772	38	10	0.25	8.472	30	8	0.4
225 KTCN	OD	*TR3*	ch01	39.278	12.948	134	13	0.325	8.712	170	9	0.45
225 KTCN	OD	*TR3*	ch02	39.278	39.732	85	40	1	19.429	53	20	1
225 KTCN	OD	*TR3*	ch03	39.278	6.417	32	6	0.15	3.232	26	3	0.15

Abbreviations: OD – right eye, OS – left eye, ROI – Region of Interest, *TR1 – central topographic region*, *TR2 – middle topographic region*, *TR3 – peripheral topographic region.*

3. SUPPLEMENTARY FIGURE LEGENDS

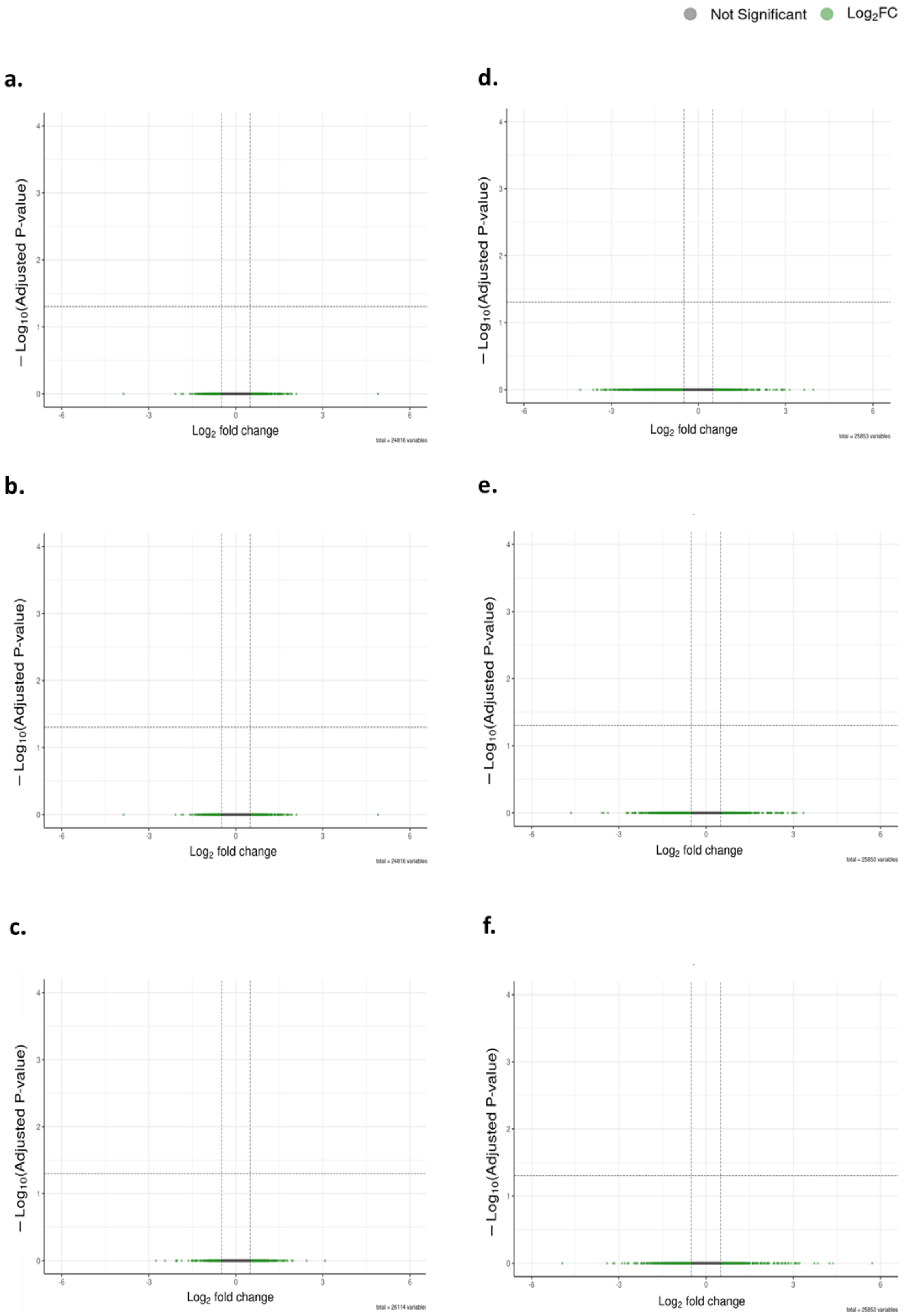

Supplementary Figure S1. Volcano plot of differentially expressed genes (DEGs). Visualization of results of the analysis of pairs of patients with KTCN (*n*=16) for (**a**) *central topographic region*, (**b**) *middle topographic region*, and (**c**) *peripheral topographic region* of corneal epithelium (CE). Visualization of results of the analysis of pairs of control individuals (*n*=6) for (**d**) *central topographic region)*, (**e**) *middle topographic region*, and (**f**) *peripheral topographic region* of corneal epithelium (CE). The green dots denote genes fitting

only the criterion of log2 fold change (log2FC > |0.5|). Grey dots denote genes with no significant changes in expression.

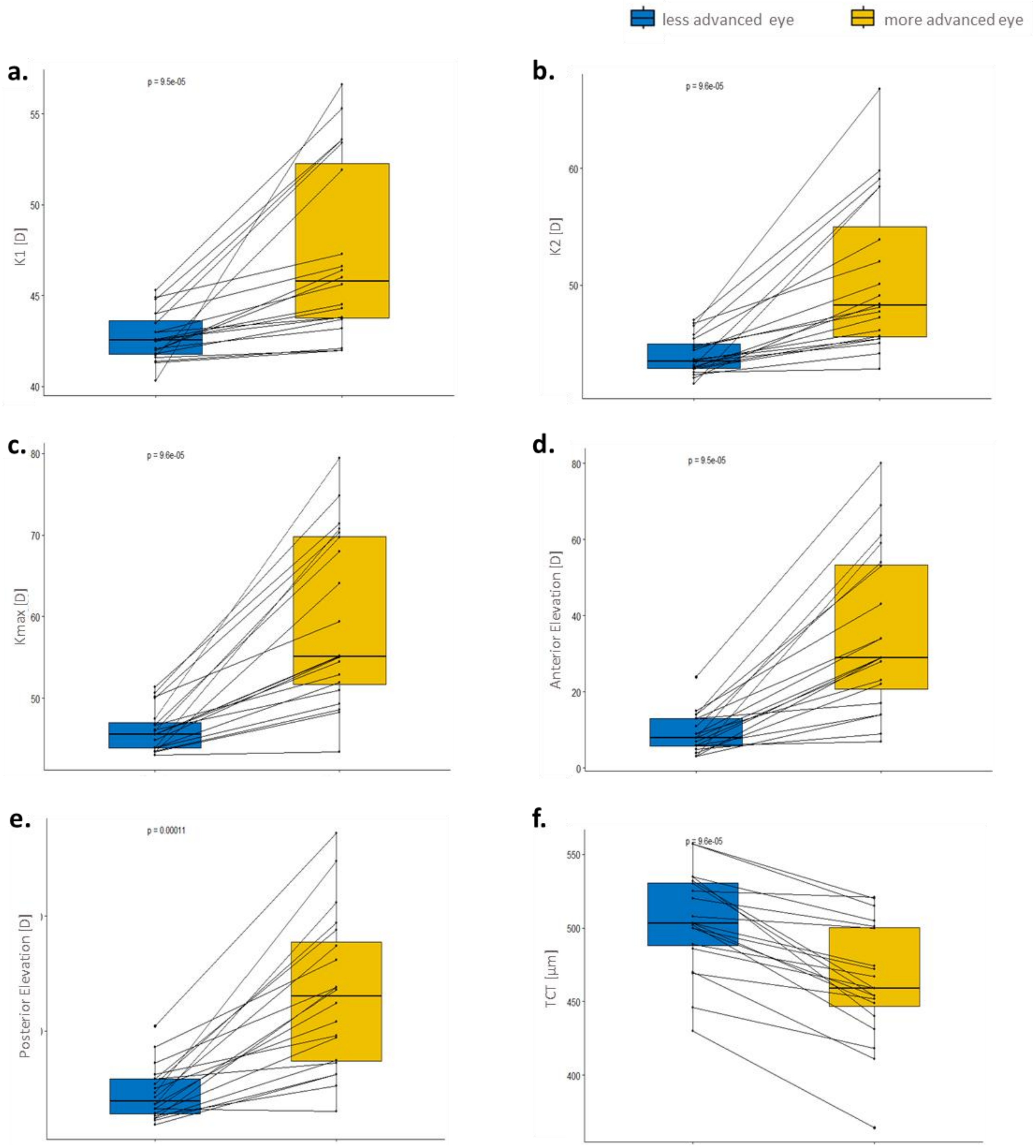

Supplementary Figure S2. Inter-eye asymmetry in clinical parameters in patients with KTCN from rediscovery studies. Comparison of (**a**) flat keratometry (K1), (**b**) steep keratometry (K2), (**c**) maximal corneal curvature (Kmax), (**d**) anterior elevation, (**e**) posterior elevation, and (**f**) thinnest corneal thickness (TCT) values in more (in yellow) and less (in blue) advanced eyes in patients with KTCN (*n*=20 pairs in rediscovery studies). The p-values of the two-tailed Wilcoxon signed-rank test are presented.

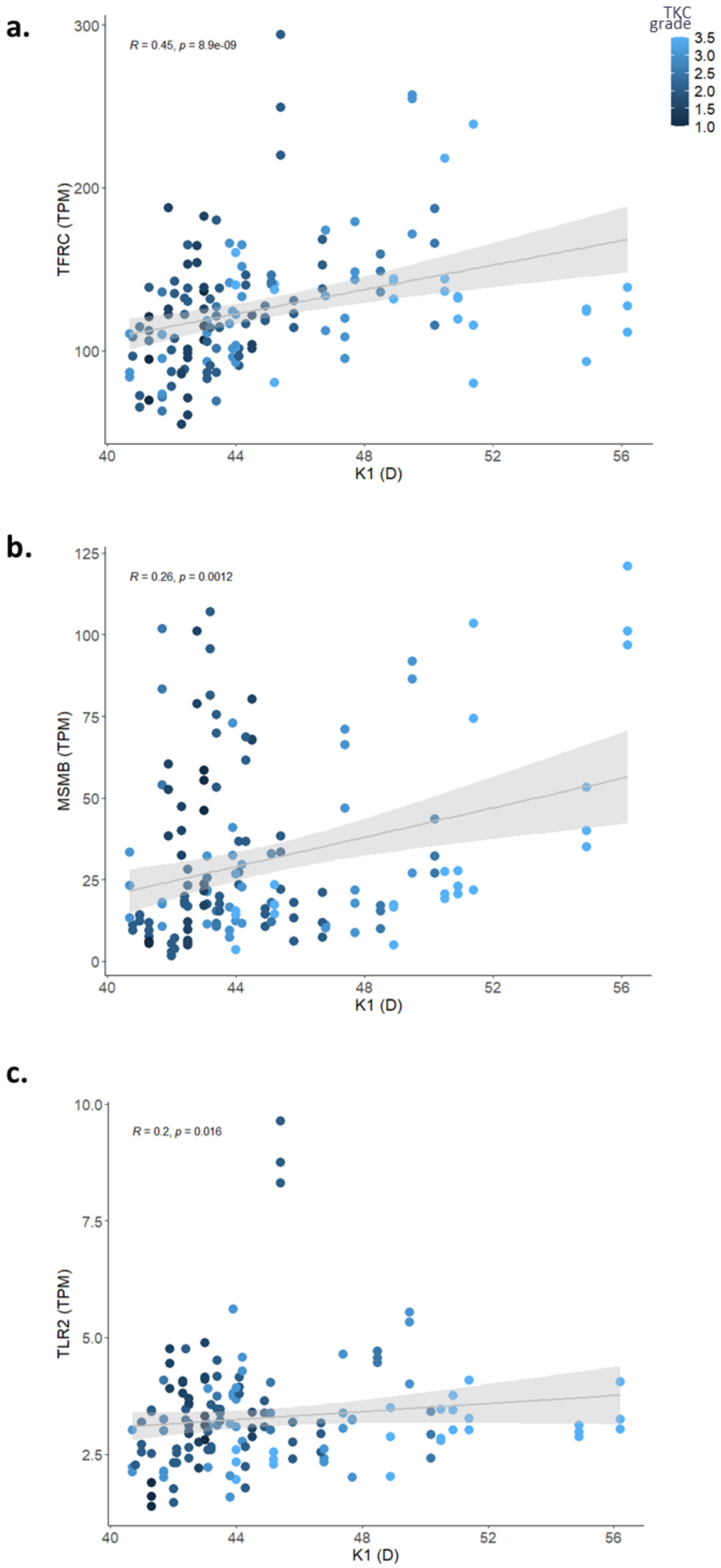

Supplementary Figure S3. Representative results of correlation analysis between the expression of *TFRC*, *MSMB*, *TLR2*, and clinical data. The Spearman correlation was calculated (**a**) between expression level of *TFRC* (in transcripts per million, TPM) in particular *topographic region* of CE and flat keratometry (K1, in [D]); (**b**) between expression level of *MSMB* (in TPM) in particular *topographic region* of CE and K1 (in [D]); and (**c**) between expression level of *TLR2* (in TPM) in particular *topographic region* of CE and K1 (in [D]) of 50 patients with KTCN. Color scale represents the Topographic Keratoconus Classification (TKC) grades, with the light blue indicating more advanced KTCN.

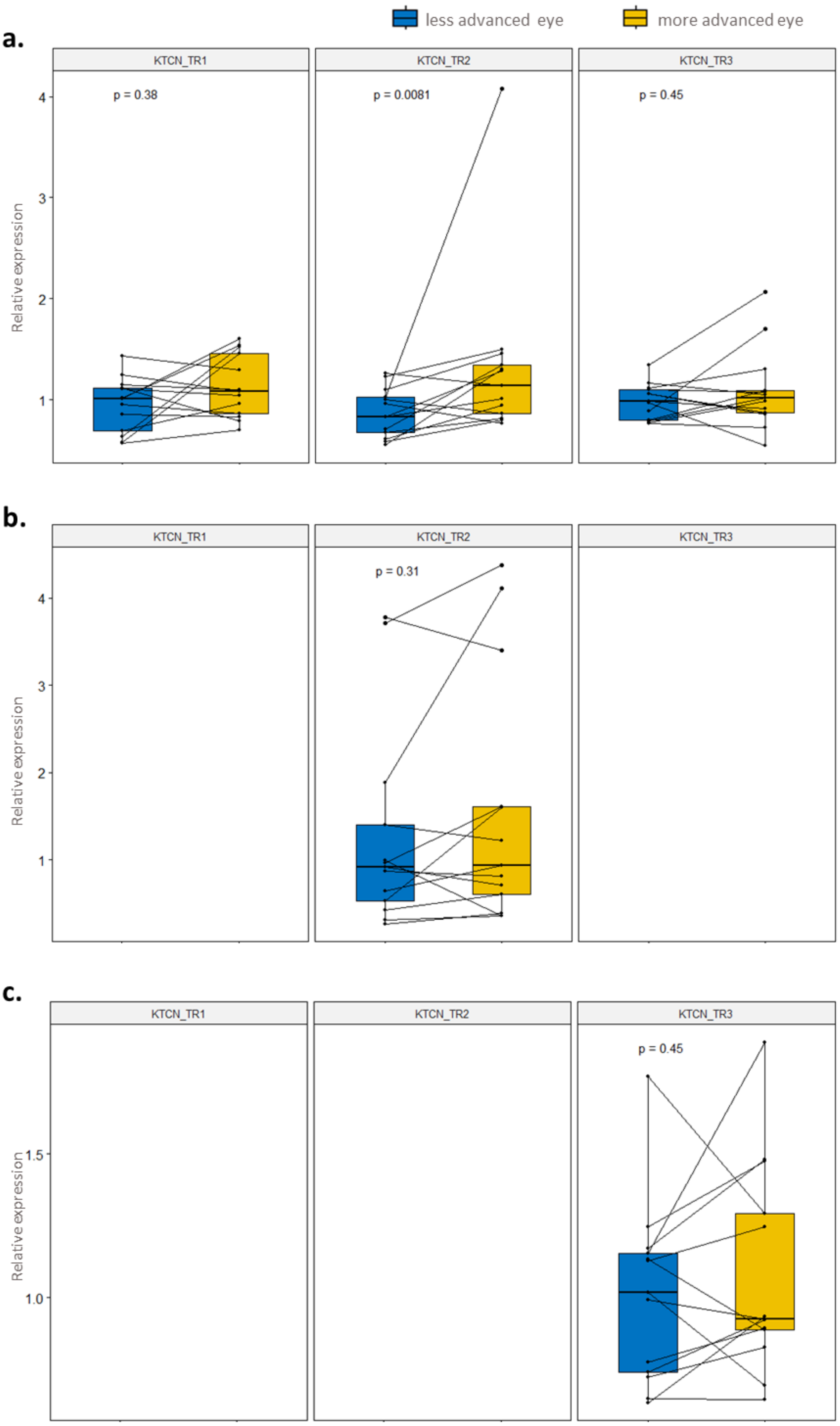

Supplementary Figure S4. The relative expression of (a) *TFRC*, (b) *MSMB*, and (c) *TLR2* in the pairs of CE samples from KTCN rediscovery group using RT-qPCR. More (in yellow) and less (in blue) advanced eyes were assigned in each eye pair of patients with KTCN (*n*=12 pairs). Relative gene expression levels were normalized to *UBC*, *LDHA*, and *RPL4* transcript levels using the comparative CT method. The p-values of paired samples t-test are presented. Abbreviation *TR1* refers to the *central topographic region*, *TR2* to the *middle topographic region*, and *TR3* to the *peripheral topographic region*.

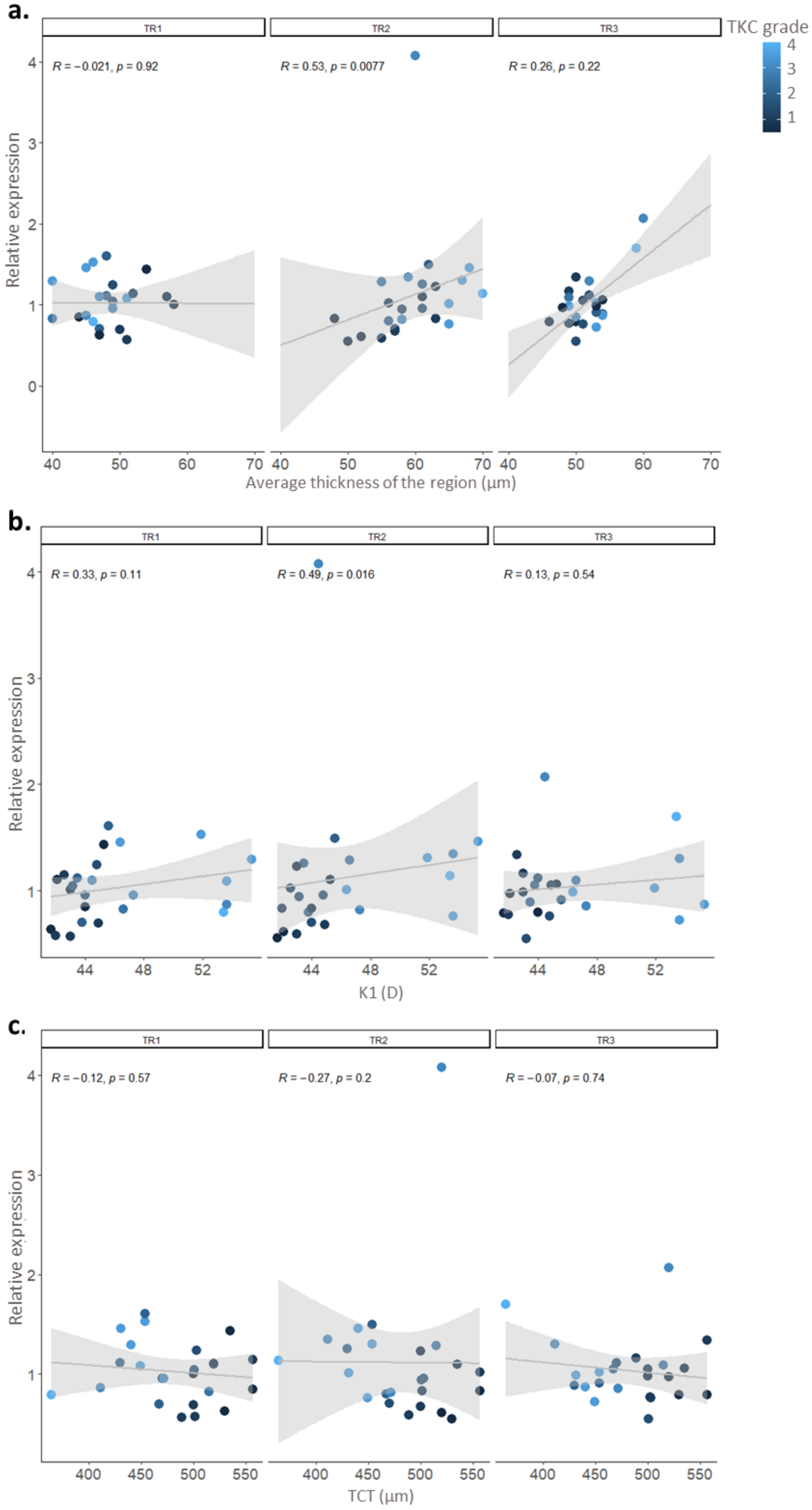

Supplementary Figure S5. The correlation between the expression of *TFRC* and clinical data. The Spearman correlation was calculated between relative expression of *TFRC* in particular *topographic regions* of corneal epithelium (CE) and (**a**) average *topographic region* thickness (in [µm]), (**b**) flat keratometry (K1,

in [D]), and (**c**) thinnest corneal thickness (TCT, in [μm]) of 12 patients with KTCN from rediscovery group (n=12 pairs). Color scale represents the Topographic Keratoconus Classification (TKC) grades, with the light blue indicating more advanced KTCN. The Spearman correlation coefficient values and p-values are presented. Abbreviation *TR1* refers to the *central topographic region*, *TR2* to the *middle topographic region*, and *TR3* to the *peripheral topographic region*.

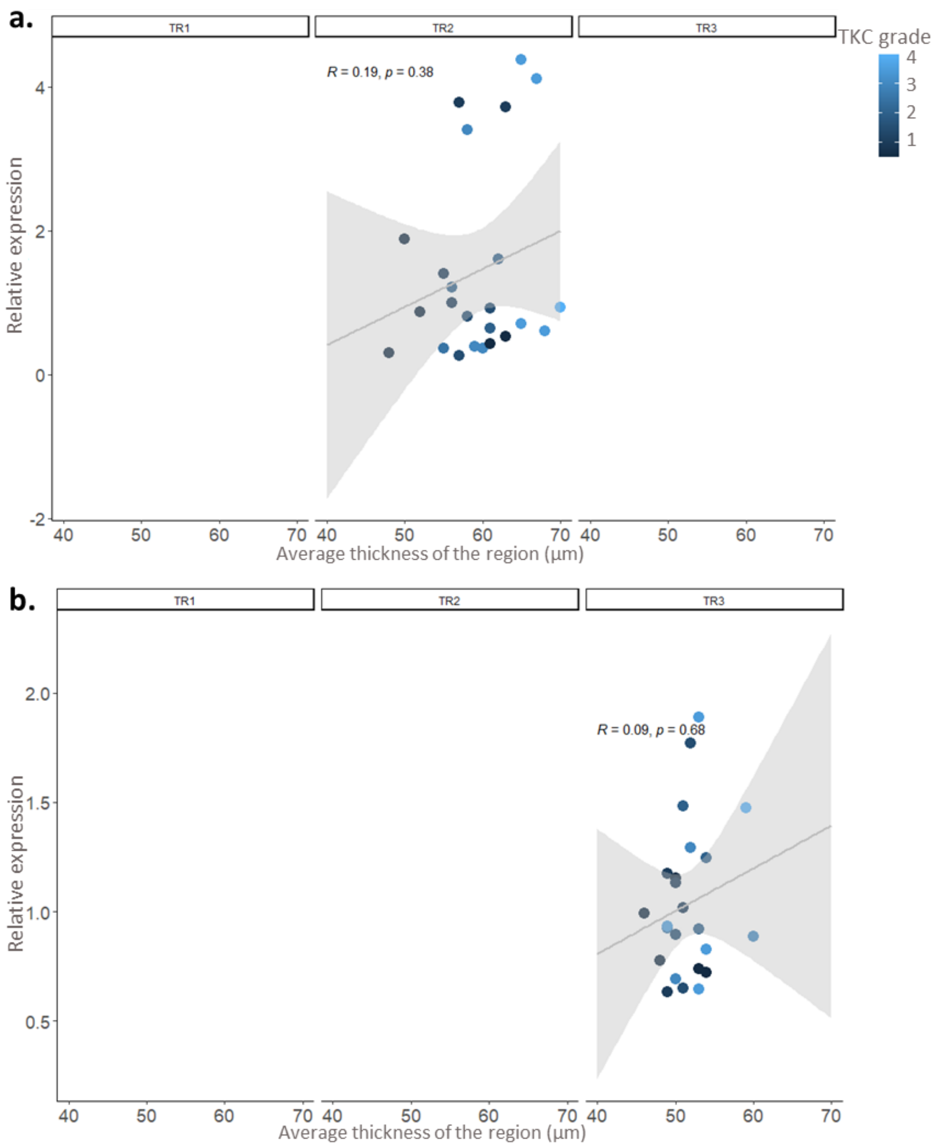

Supplementary Figure S6. The correlation between the expression of *MSMB* and *TLR2*, and clinical data. The Spearman correlation was calculated (**a**) between relative expression of *MSMB* in *middle topographic region* of CE and average *topographic region* thickness (in [µm]); and (**b**) between relative expression of *TLR2* in *peripheral topographic region* of CE and average *topographic region* thickness (in [µm]) of 13 patients with KTCN from rediscovery group (n=26). Color scale represents the Topographic Keratoconus Classification (TKC) grades, with the light blue indicating more advanced KTCN. The Spearman correlation coefficient values and p-values are presented. Abbreviation *TR1* refers to the *central topographic region*, *TR*2 to the *middle topographic region*, and *TR3* to the *peripheral topographic region*.

4. REFERENCES

Ge, S.X., Jung, D., Yao, R., 2020. ShinyGO: a graphical gene-set enrichment tool for animals and plants. Bioinformatics 36, 2628–2629. https://doi.org/10.1093/bioinformatics/btz931

JASP Team, 2022, 2022. JASP Team (2022). JASP (Version 0.16.3)[Computer software]. JASP.

Law, C.W., Alhamdoosh, M., Su, S., Dong, X., Tian, L., Smyth, G.K., Ritchie, M.E., 2018. RNA-seq analysis is easy as 1-2-3 with limma, Glimma and edgeR. F1000Res 5, 1408. https://doi.org/10.12688/f1000research.9005.3

Ritchie, M.E., Phipson, B., Wu, D., Hu, Y., Law, C.W., Shi, W., Smyth, G.K., 2015. limma powers differential expression analyses for RNA-sequencing and microarray studies. Nucleic Acids Res 43, e47. https://doi.org/10.1093/nar/gkv007

Schindelin, J., Arganda-Carreras, I., Frise, E., Kaynig, V., Longair, M., Pietzsch, T., Preibisch, S., Rueden, C., Saalfeld, S., Schmid, B., Tinevez, J.-Y., White, D.J., Hartenstein, V., Eliceiri, K., Tomancak, P., Cardona, A., 2012. Fiji: an open-source platform for biological-image analysis. Nat Methods 9, 676–682. https://doi.org/10.1038/nmeth.2019
